# Contract farming and rural transformation: Evidence from a field experiment in Benin^☆^

**DOI:** 10.1016/j.jdeveco.2021.102626

**Published:** 2021-06

**Authors:** Aminou Arouna, Jeffrey D. Michler, Jourdain C. Lokossou

**Affiliations:** a Africa Rice Center (AfricaRice), Bouake, Cote d’Ivoire; b Department of Agricultural and Resource Economics, University of Arizona, Tucson, USA; c International Crop Research Institute for the Semi-Arid Tropics (ICRISAT), Bamako, Mali

**Keywords:** Structural transformation, Contract farming, RCT, Rice, Sub-Saharan Africa

## Abstract

Contract farming has emerged as a popular mechanism to encourage vertical coordination in developing country agriculture. Yet, there is a lack of consensus on its ability to spur structural transformation in rural economies. We present results from a field experiment on contract farming for rice production in Benin. While all contracts have positive effects on welfare and productivity measures, we find that the simplest contract has impacts nearly as large as contracts with additional attributes. This suggests that once price risk is resolved through the offer of a fixed-price contract, farmers are able to address other constraints on their own.

## Highlights

• We describe evidence from the first RCT on contract farming in a developing country.
• All contracts have positive effects on welfare and productivity measures.
• The simplest contract has impacts nearly as large as contracts with additional attributes.
• Results suggests that once price risk is resolved farmers are able to address other constraints on their own.

## 1 Introduction

Structural transformation is a fundamental challenge in economic development and key to overcoming food insecurity and poverty for the millions of households that work in agriculture (Timmer, 1988). An extensive literature demonstrates the variety of constraints that hinder the transition of rural farms from subsistence to commercial production. Among these are price uncertainty (Saha, 1994; Bellemare et al., 2013), access to credit (Berg, 2013; McArthur and McCord, 2017), and a lack of technical knowledge (Sherlund et al., 2002; Hanna et al., 2014). These constraints affect input demand, as well as yields, sales, and income, contributing to the perpetuation of the agrarian status quo.

Vertical coordination has the potential for fostering structural transformation of rural economies (Dixit, 2004; Acemoglu et al., 2009; Barrett et al., 2017). In recent years, contract farming has emerged as a popular mechanism to encourage such vertical coordination (Barrett et al., 2012; Wang et al., 2014b; Otsuka et al., 2016; de Janvry et al., 2017). Farm production contracts can shift risk and the need for initial capital from small farming households to medium and large processors who are better able to manage these issues. In return, firms secure a stream of quality inputs for processing. While many see contract farming as a way to spur rural structural transformation and growth in local economies, the view is far from universal.^1^ As Bellemare and Bloem (2018) and Ton et al. (2018) point out, one reason for the lack of consensus on the impacts of contract farming in developing countries is that, up till now, studies have relied exclusively on observational data, and many have been limited to cross-sectional data.

We present results from the first field experiment on contract farming in a developing country context.^2^ Working with a rice processing firm in Benin, we implement a randomized control trial (RCT) in which we offer rice production contracts to a random subset of smallholder farmers. We find that contract farming has a positive and significant impact on a number of different measures of rural transformation, such as scale, productivity, and commercial orientation. Relative to the control, contract farming households increase their area planted to rice by 23 percent, increase yields by 29 percent, more than double the share of output sold in the market, and increase per capita income by half.

Given these findings, it is particularly important to understand the contract attributes driving the results, not only in terms of production outcomes but also for household income. To accomplish this, we further randomize treated farmers into one of three types of production contracts. The first is a fixed-price contract in which the processor offers farmers a guaranteed price for their rice production.^3^ The second is a production-management contract in which the processor sends extension agents three to five times throughout the growing season to provide agricultural training and technical assistance. The third is an input-supply contract in which the processor provides input loans for seed and fertilizer and deducts the cost at harvest. Because of our implementing partner’s finite resources, we randomize within the primary experiment whether the contract 1) provides a price guarantee, 2) combines extension training with the price guarantee, or 3) provides input loans in addition to the extension training and price guarantee.

We find that the magnitude of the treatment effect on area planted to rice and the share of output sold in the market varies significantly depending on contract attributes. However, we find no significant differences in the magnitude of the treatment effect on rice yields or income per capita based on contract attributes. In fact, the fixed-price contract frequently results in treatment effects on area planted, yield, and income per capita that are statistically indistinguishable from the more complex (and costlier) contracts that provide extension training and/or input loans. This suggests that once price risk is resolved, farmers can, on their own, address issues of technical efficiency and capital constraints.

Our study contributes foremost to the empirical literature on incentives in agricultural production contracts. Numerous empirical studies in developing countries analyze correlates with the decision to participate in contract farming (Barrett et al., 2012). A rapidly growing literature also seeks to identify the effect of contracts and contract attributes on production and income, yet the endogeneity of contract choice is a potential source of bias in these findings.^4^ We provide the first experimental evidence on the impact of contract farming in a developing country. Additionally, we assess the impact of different contract attributes on production decisions and the realization of farm income. Our results show that contract farming has a positive impact on several measures of farm production and income. Furthermore, we focus our experiment on contract farming of a staple crop. The majority of the literature on contracting farming focuses on high-value and specialty crops.^5^ Unlike specialty crops, the margins, and therefore the incentives, for staple crop cultivation are small (Otsuka et al., 2016). This suggests that our results should not only be generalizable to contract farming for other staple crops but may be a lower bound on the impacts that contract farming could have on higher value, higher margin specialty crops.

More broadly, this study contributes to the theoretical and empirical literature that views the persistence of the agrarian status quo in developing countries as a mechanism design problem. This literature goes back to Stiglitz (1974), North (1984), Williamson (1994), and Dixit (2004). Recent empirical examples include Casaburi and Willis (2018), who examine liquidity constraints within insurance contracts in Kenya, Blouin and Macchiavello (2019), who consider the role of counterparty risk in production contracts in Rwanda, Burchardi et al. (2019), who investigate the incentive effect of tenancy contracts in Uganda, and Casaburi and Macchiavello (2019), who study commitment devices for incomplete contracts in Kenya. We show that careful design of agricultural production contracts can relax constraints and reduce risk for farming households, allowing them to commercialize and contribute to the process of rural transformation.

## 2 Context and experimental design

### 2.1 Contract farming

When farmers are risk averse they fail to equate the expected marginal value of an input to its price. The distortion to optimal input use can depend on a number of factors, including price risk, technical inefficiency, and capital constraints. If Farmer A faces risk regarding the price at which she may sell her output compared to an identical Farmer B facing no price risk, Farmer A will use less of an input relative to Farmer B. Similarly, technical inefficiency as well as a binding capital constraint, increases the size of the distortion in optimal input use, resulting in under use of the input relative to identical farmers who are not capital constrained and/or are technically efficient. Consequently, anything that depends on input demand functions, such as input productivity, output supply, and profitability, will also be affected by price risk, technical inefficiency, and capital constraints.

Contract farming is a mechanism that can reduce or eliminate these distortions to optimal input demand. Mighell and Jones (1963) classify farming contracts into three categories: 1) fixed-price contracts, which describe the terms of the sales transaction with regard to price, quantity, timing, and product attributes; 2) production-management contracts, which specify the way the commodity is to be grown, such as the planting density, use of pesticides, and timing of harvest; and 3) input-supply contracts, in which the buyer provides inputs, often on credit. Each type of contract addresses a different source of risk or different constraints, though all provide a guaranteed market for the crop. Fixed-price contracts, by guaranteeing a price, insulate producers from price risk. Production-management contracts, by defining the optimal production process, reduce technical inefficiency. Input-supply contracts, by providing inputs at the start of the season, relax the credit or liquidity constraints faced by farmers. It is an empirical, and context dependent, question regarding which contract attributes will be most effective in reducing risks and easing constraints that hinder commercialization and ultimately rural transformation.

### 2.2 Study setting

In the last decade, rice production in Benin has increased rapidly, though production remains concentrated in three departments: Atakora, Alibori, and Collines (Maertens and Vande Velde, 2017). We work with smallholder rice farmers in four municipalities with Collines, which the Ministry of Agriculture, Livestock, and Fisheries identifies as having substantial potential for producing rice (MAEP, 2011). In this region, rice is grown in rainfed lowlands, meaning it requires no irrigation and it does not compete for space with existing crops such as cotton, maize, or cassava. Rice is planted in the second half of the year and harvested in December or January, allowing for only one growing season per year. Most rice is produced for home consumption, but the average farmer in our baseline data sells around a quarter of their harvest into the market. Buyers are typically collectors or traders that bulk rice and sell it to a processor, or the processor can buy directly from the farm via the spot market or a production contract.

The experiment was implemented in collaboration with *Entreprises de Services et Organizations de Producteurs de Bante* (ESOP), a private rice processing and marketing firm that has experience in purchasing rice through farming contracts. The organization purchases paddy rice, which it then de-husks, polishes, sorts, and packages. The product is marketed as local, high quality rice, priced at a premium compared to other domestic rice, which is typically sold in bulk as an undifferentiated product. ESOP owns a milling facility with the capacity to process 300,000 ​kg of paddy rice per year but has consistently operated below capacity, as the spot market lacks paddy rice in sufficient quantity and of sufficient quality to meet ESOP’s demand. Because of this, ESOP offers production contracts to local smallholder farmers. Though contracts are signed with individual farmers, participants are asked to form groups of eight to ten in order to simplify the logistics of delivering inputs and collecting output at the end of the season. ESOP’s standard contract is a combination of the fixed-price, production-management, and input-supply contracts outlined in Mighell and Jones (1963). The contract offers a fixed price for rice that meets a stipulated quality threshold and includes the provision of inputs, on loan, and agricultural training and technical assistance throughout the growing season. For ESOP, the contracts are profitable but the need to raise sufficient capital to provide input loans has limited the organization’s ability to expand.

### 2.3 Experimental design and sample selection

**Fig. 1:**
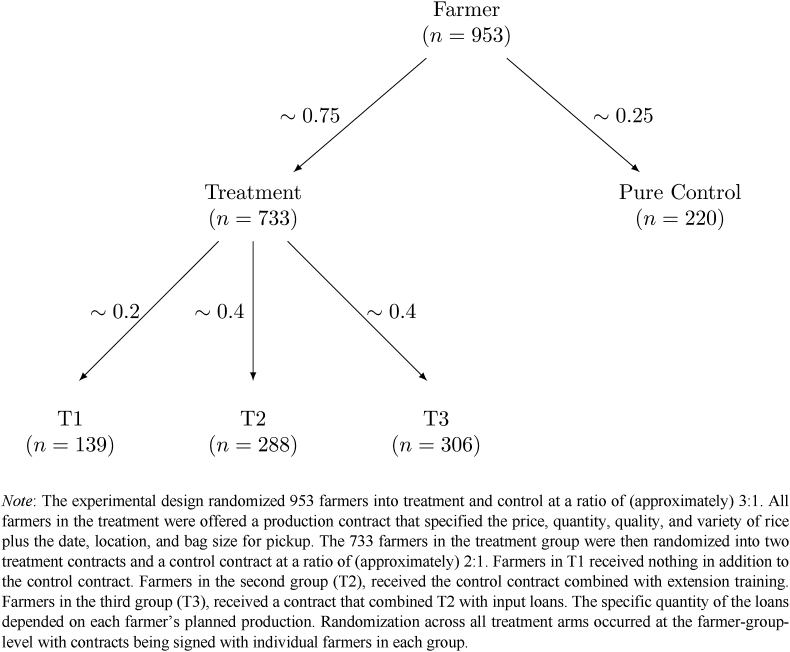
Design of experiment.

The experimental design randomly assigned farmer-groups to treatment and control at a ratio of approximately 3:1 (Fig. 1). All farmers in the treatment were offered a production contract that specified the price, quantity, quality, and variety of rice, plus the date, location, and the size of the bag the rice needed to be in for pickup after harvest. Farmers in the treatment group were then randomized into two treatment contracts and a control contract at a ratio of approximately 2:1. The specifics of each contract are described in subsection 2.4 below. Additional details and English translations of the French contracts are provided in Appendix A.

In order to select representative households within the four municipalities where ESOP works, we obtained a list of rice farmers from the National Office of Agricultural Statistics. The list is produced from a Ministry of Agriculture census of farmers that details the crops each farmer cultivates and was last updated in 2015. From the list of rice producers, we randomly selected 953 farmers to participate in the study. In June 2016, per ESOP’s operating procedure, farmers were requested to form groups of eight farmers each in order to simplify the logistics of delivering inputs (if farmers ended up being randomly assigned to receive them) and collecting rice at harvest. A baseline survey was conducted in July 2016, prior to randomization, and collected information on the 2015–16 rice growing season, along with sociodemographic characteristics.^6^ Random assignment was conducted at meetings in August 2016 with randomization done at the farmer group level to reduce spillovers and comply with ESOP’s standard procedures.^7^ Though randomization was at the group level, each farmer in a group allocated to the treatment signed their own written contract with the rice milling unit of ESOP. An endline survey conducted in January 2017 collected information on the 2016-17 growing season, as well as any changes to household characteristics.^8^ Fig. B1 in the Appendix provides a timeline of the rice growing season and the experiment.

### 2.4 Interventions

Within the treatment arm, we provided three types of contracts aligning to those discussed in Mighell and Jones (1963). However, all three contracts shared a number of clauses. For all contracts, the sales price was fixed at 150 CFA per kg.^9^ The market price typically ranges from 110 to 170 CFA per kg, before accounting for the buyer (collectors, traders, or processors) and the place of sale (farm gate, market, or mill). Additionally, all contracts specified the variety of rice to be grown (IR841, a common variety in the region), the minimum level of quality (a threshold for the percentage of impurities present, such as pebbles or other debris), the date and location where the rice would be collected, and the size of bags in which the rice must be delivered (80–100 ​kg bags). The contracts also specified how much rice the farmer would deliver to ESOP, the quantity being determined by the farmer based on how much land area the farmer planned to cultivate with rice. Finally, all contract defined how breach of contract was to be resolved. Contracts were signed by an ESOP representative with individual farmers in the presence of group members, and were witnessed as well. All contracts were designed to be revenue equivalent in terms of price and measured inputs. Additional details are provided in the Appendix.

The first treatment (T1) provided a fixed-price contract to farmers which specified the price at which ESOP was willing to purchase rice. This “control” contract specified nothing more than those elements outlined above (quantity, quality, variety, and delivery date and location), which are standard elements in any contract for agricultural production (Otsuka et al., 2016).^10^

The second treatment (T2) combined a fixed-price contract with a production-management contract. The contract included all the attributes of T1, including the same price, and added a stipulation that throughout the season farmers would receive between three and five visits from ESOP extension agents. The extension agents advised the farmers on good agricultural practices, in regards to planting, the application of fertilizer, the tending of rice at its various stages of growth, and post-harvest handling.

The third treatment (T3) added an input-supply contract to a fixed-price and production-management contract. The contract included all the attributes of T2 and added the provision of inputs, on loan, from ESOP. The contract stipulated the amount of seed (45 ​kg/ha) and fertilizer (150 ​kg/ha) to be provided as well as the price for the inputs. At the end of the season, the total cost of inputs provided would be deducted from the price paid to the farmers. ESOP did not charge any interest on the loans but only recouped the cost of the inputs.^11^

**Fig. 2:**
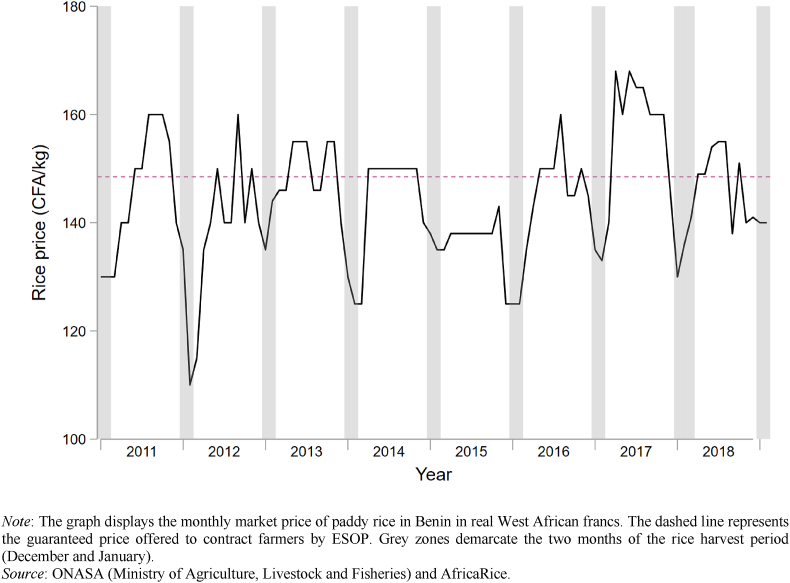
Historic rice price.

*Source*: ONASA (Ministry of Agriculture, Livestock and Fisheries) and AfricaRice.

It is natural to wonder about the degree to which farmers in our sample are subject to price risk and credit constraints, and their level of efficiency in rice production. Regarding price risk, we obtained monthly data from the Ministry of Agriculture for the period 2010–2019. Fig. 2 graphs the monthly price and overlays gray bars to designate the harvest season. Prices show a high degree of seasonality, with prices falling by as much as 30 percent in the periods immediately after harvest. To this graph is added a dashed line that represents the guaranteed price provided by the contracts. The price of 150 CFA offered by ESOP is slightly above the eight-year monthly average of 144 CFA (equivalent to a US$0.01 difference per kg).^12^ While the contract price is typically below the seasonal high, in each of the last eight years it has been above the seasonal low, which occurs at harvest.

Evidence on credit constraints and production efficiency is equally strong. As part of the baseline survey, we asked farmers about the credit they received from both formal and informal sources. Seventy-eight percent of farmers reported that they lacked access to credit. For those who did have access, credit utilization was low. While the mean dollar amount received was US$99.36 the median value was zero. Levels of rice production were also low. AfricaRice estimates that rice producers in Benin should be able to average 2000 ​kg per hectare in rainfed production and 3500 ​kg per hectare under irrigation. According to the most recent data available from FAOSTAT, which aggregates rainfed and irrigated rice, average rice production in Benin was 3140 ​kg per hectare in 2014. In our baseline data, the average yield was 822, with a median of 400. While these values are simply descriptive, they do offer *prima facie* evidence of farmers in the sample operating well below the technical efficiency frontier.

## 3 Data

### 3.1 Baseline data and measurement

In the baseline survey we asked farmers about their previous experience with contract farming. Contract farming was relatively well known among participants, with 60 percent aware of the existence of contract farming and 74 percent of these having engaged in at least one contract for crop production (Table 1). The majority of these contracts, 67 percent, were written agreements. Around 79 percent of farmers had engaged in a contract that set the price while 74 percent of farmers had engaged in a contract that set the quantity. The most common type of contract (83 percent) was one that included terms governing quality. About 70 percent of contracts included input loans and 36 percent included some aspect of production-management. Note that most farming contracts were for cotton, which is a cash crop in the surveyed area.

**Table 1:** Attributes of existing contract farming arrangements.

		Number	Percentage
Awareness of contract farming		567	59.50
Experience in contract farming		419	73.90
Type of contract	Oral	138	32.94
	Written	281	67.06
Agreement on price		334	79.71
Agreement on quality		346	82.58
Agreement on quantity		313	74.70
Technical training		151	36.04
Credit	In-kind credit	254	60.62
	In-cash credit	43	10.26

*Note*: Table displays number of farmers and percentage of farmers in the data set that responded in the affirmative to questions regarding their awareness of and experience with contract farming.

We investigate contract farming’s impact on four key outcome variables. Rice area is measured as the total land in hectares area cultivated with rice. Yield is measured as the total amount of rice harvested in kg divided by area cultivated. Market participation is measured as the share of harvested rice that was sold, either to a rice processor, such as a parboiler, or into the market for paddy rice.^13^ Rice that is not marketed is either kept for consumption or saved for seed. Household income is measured as the sum of income from rice, from other crop production and livestock sales, and any income from non-farm activity. Non-farm activity consists of salaried employment, small time trading, wage labor, and remittances. Crop production not sold is valued at market prices, though we do not value livestock consumed by the household or on-farm household labor as we lack detailed data on these inputs. Total income is then divided by household size to arrive at income per capita.

Our analysis relies on self-reported measures for all variables. In the last couple of years, a number of studies using observational data have shown that several stylized facts in the development literature, such as the inverse productivity-size relationship, are in reality a result of bias introduced by non-classical measurement error (Abay et al., 2019). The lack of objective measures in our experiment is not ideal, though in our context measurement error likely introduces only noise, not bias, into our estimates. This is because any measurement error in our self-reported outcome variables will be orthogonal to our randomized treatment, absent any Hawthorne effects.^14^ If farmer recall of any of our outcome variables is better remembered when under contract, Hawthorne effects may exist. Such effects are not a given, though it is possible that farmers with contracts report yields with less measurement error than those not under contract. To check whether those assigned to treatment remember better than those in the control, we calculate the coefficient of variation across groups at endline. If Hawthorne effects do exist through focusing treated farmers on outcomes, we would expect less variation in treated outcomes. As can be seen from Table B1 in the Appendix, coefficients of variation are similar for those in the control compared to those in the treatment. We take this as evidence that any measurement error in our variables of interest is classical in nature, being uncorrelated with random assignment to treatment.

### 3.2 Balance

Table 2 presents descriptive statistics for our dependent and independent variables for the different treatment groups.^15^ The first four columns of the table present means and standard deviations for each treatment and the control at baseline. The final six columns of the table present coefficients and standard errors from OLS regressions comparing farmers across treatments and with the control. For each cell, we regress the variable of interest (row) on an indicator of treatment status (column). Standard errors are clustered at the farmer-group-level, which is our unit of randomization.

**Table 2:** Baseline summary statistics and balance test.

	Control (n ​= ​220)	Treatment [T] (n ​= ​733)	Price [T1] (n ​= ​139)	Extension & price [T2] (n ​= ​288)	Input loans, extension, & price [T3] (n ​= ​306)	Differences in treatment status within groups						
						[T-C]	[T1-C]	[T2-C]	[T3-C]	[T2-T1]	[T3-T1]	[T3-T2]
Rice area (ha)	0.629 (0.751)	0.855 (1.969)	0.636 (0.770)	1.050 (2.848)	0.772 (1.157)	0.227∗ (0.105)	0.007 (0.086)	0.421∗ (0.203)	0.144 (0.117)	0.414∗ (0.205)	0.136 (0.120)	−0.278 (0.219)
Yield (kg/ha)	819.2 (1494)	903.1 (1523)	832.9 (1742)	980.1 (1589)	862.5 (1345)	83.91 (172.4)	13.72 (264.7)	160.9 (240.8)	43.32 (207.3)	147.2 (306.1)	29.60 (280.2)	−117.6 (257.7)
Market participation (%)	28.83 (38.16)	41.12 (41.71)	27.40 (37.62)	45.08 (41.55)	43.64 (42.47)	12.30∗ (5.037)	−1.432 (5.567)	16.26∗ (7.394)	14.82∗ (6.684)	17.69∗ (7.838)	16.25∗ (7.166)	−1.440 (8.655)
Income per capita (US$)	234.1 (321.5)	218.7 (349.6)	231.6 (478.5)	224.4 (336.7)	207.6 (287.9)	−15.31 (32.99)	−2.446 (80.50)	−9.691 (44.97)	−26.44 (39.09)	−7.244 (87.31)	−24.00 (84.38)	−16.75 (51.52)
Household size	8.836 (4.355)	8.116 (3.756)	7.712 (3.602)	8.243 (3.755)	8.180 (3.824)	−0.720∗ (0.362)	−1.124∗ (0.547)	−0.593 (0.539)	−0.657 (0.469)	0.531 (0.685)	0.468 (0.631)	−0.063 (0.623)
Age of household head (years)	40.56 (8.972)	41.36 (10.59)	41.76 (10.21)	42.09 (10.67)	40.50 (10.66)	0.810 (0.858)	1.201 (1.244)	1.539 (1.174)	−0.055 (1.111)	0.338 (1.473)	−1.255 (1.422)	−1.594 (1.360)
Male headed household (=1)	0.564 (0.497)	0.561 (0.497)	0.626 (0.486)	0.497 (0.501)	0.592 (0.492)	−0.003 (0.056)	0.062 (0.082)	−0.067 (0.071)	0.028 (0.064)	−0.129 (0.088)	−0.034 (0.081)	0.095 (0.071)
Exp. Producing rice (years)	8.195 (3.593)	8.748 (5.532)	7.748 (5.593)	9.788 (5.922)	8.222 (4.955)	0.552 (0.544)	−0.447 (1.106)	1.593 (0.886)	0.027 (0.656)	2.040 (1.378)	0.474 (1.241)	−1.566 (1.049)
Primary education (=1)	0.114 (0.318)	0.098 (0.298)	0.086 (0.282)	0.118 (0.323)	0.085 (0.279)	−0.015 (0.021)	−0.027 (0.032)	0.004 (0.024)	−0.029 (0.023)	0.032 (0.031)	−0.001 (0.030)	−0.033 (0.021)
Farming is main activity (=1)	0.918 (0.275)	0.928 (0.259)	0.950 (0.219)	0.924 (0.266)	0.922 (0.269)	0.010 (0.024)	0.031 (0.036)	0.005 (0.029)	0.003 (0.034)	−0.026 (0.037)	−0.028 (0.042)	−0.002 (0.036)
Training in rice production (=1)	0.527 (0.500)	0.505 (0.500)	0.194 (0.397)	0.628 (0.484)	0.529 (0.500)	−0.022 (0.062)	−0.333∗∗∗ (0.061)	0.101 (0.084)	0.002 (0.089)	0.434∗∗∗ (0.090)	0.335∗∗∗ (0.095)	−0.099 (0.111)
Member of farm association (=1)	0.968 (0.176)	0.965 (0.185)	0.906 (0.292)	0.979 (0.143)	0.977 (0.150)	−0.004 (0.015)	−0.062 (0.038)	0.011 (0.018)	0.009 (0.014)	0.073 (0.039)	0.071 (0.037)	−0.002 (0.018)

*Note*: The first five columns report means of the data at baseline with standard deviations in parentheses. The final seven columns report coefficients and standard errors from OLS regressions of the variables of interest or the covariates on treatment status within different groups. Standard errors clustered at the farmer-group-level are in parentheses (*∗∗∗*p<0.01*, ∗∗*p<0.05*, ∗*p<0.10).

Farmers randomly assigned to the pooled treatment had a significantly larger area planted to rice prior to the experiment. Control farmers tended to plant 0.62 ​ha to rice while treatment farmers tended to plant 0.86 ​ha to rice. Average yields in the baseline vary between 820 and 980 ​kg per hectare but with large standard deviations and no differences across treatment and control. For market participation we see some differences across multiple treatments. Farmers randomly assigned to the control and T1 sold about 30 percent of their pre-experiment rice production into the market. By comparison, farmers randomly assigned to the other two contracts sold about 45 percent of their pre-experiment rice production in the market. Despite this greater share of market participation prior to the experiment, per capita income was no different across the four groups, with average income being about $220 per person. To control for where we lack baseline balance in our dependent variables, we use an Analysis of Covariance (ANCOVA) estimator.

Among our control variables, the average farm household had eight members with the head of the household aged 40 years. Around 60 percent of households were male headed with the household head having grown rice for around eight years. Only ten percent of household heads had even a primary education while 90 percent of households listed farming as their primary business or activity. Nearly 100 percent of household heads were members of a farming association. Farmers varied in whether or not they had participated in training on rice production. While only 20 percent of farmers randomized into T1 had participated in training, around 55 percent of farmers in the control and other two treatments had training in rice production.

In addition to checking balance by examining the correlation between treatment assignment and each individual outcome variable or household characteristic, we also regress treatment assignment on the complete set of outcome variables and covariates. Table 3 presents the results from these six regressions as well as the F-statistic from a test of joint significance. In general, both of our balance checks suggest that our randomization was effective, though differences do exist across a small number of variables. Where the F-statistic is significant, this is typically due to significant differences in control variables, such as past participation in rice training, and not due to differences in the outcome variables. In general, differences do not appear to be indicative of systematic variation across multiple treatments and we employ an empirical strategy that allows us to control for where differences do exist.

**Table 3:** Balance test across treatments.

	Differences in treatment status within groups					
	[T1-C]	[T2-C]	[T3-C]	[T2-T1]	[T3-T1]	[T3-T2]
Rice area (ha)	0.019 (0.013)	0.002 (0.005)	0.011 (0.015)	0.003 (0.007)	−0.031 (0.028)	−0.022∗∗ (0.008)
Yield (kg/ha)	−0.000 (0.000)	−0.000 (0.000)	−0.000 (0.000)	0.000 (0.000)	−0.000 (0.000)	−0.000 (0.000)
Market participation (%)	−0.001 (0.000)	−0.000 (0.001)	0.000 (0.001)	0.001 (0.001)	0.001 (0.001)	0.000 (0.001)
Income per capita (US$)	−0.000 (0.000)	−0.000 (0.000)	−0.000 (0.000)	0.000 (0.000)	−0.000 (0.000)	−0.000 (0.000)
Household size	−0.001 (0.003)	−0.003 (0.005)	−0.003 (0.004)	0.013 (0.007)	0.014 (0.007)	−0.002 (0.008)
Age of household head (years)	0.003 (0.001)	0.000 (0.001)	−0.000 (0.001)	−0.004 (0.002)	−0.003 (0.003)	−0.003 (0.003)
Male headed household (=1)	−0.048 (0.027)	−0.007 (0.036)	0.012 (0.031)	−0.122∗∗ (0.039)	−0.016 (0.037)	0.149∗∗ (0.051)
Experience producing rice (years)	0.013∗∗ (0.005)	0.020∗∗∗ (0.005)	0.008∗ (0.003)	0.003 (0.005)	−0.004 (0.008)	−0.005 (0.007)
Primary education (=1)	0.028 (0.027)	0.031 (0.022)	−0.041 (0.025)	0.036 (0.039)	−0.070 (0.069)	−0.115∗ (0.047)
Farming is main activity (=1)	−0.066 (0.066)	−0.031 (0.027)	0.023 (0.027)	0.001 (0.100)	−0.035 (0.107)	0.025 (0.080)
Training in rice production (=1)	−0.137∗∗ (0.048)	−0.073 (0.060)	−0.177∗∗ (0.054)	0.272∗ (0.103)	0.197∗ (0.090)	−0.225∗ (0.086)
Member of farm assoc. (=1)	−0.022 (0.022)	−0.046 (0.051)	−0.057 (0.054)	−0.036 (0.039)	0.104 (0.086)	0.091 (0.148)
Observations	359	508	526	427	445	594
F-test of joint significance	1.93∗	1.44	1.19	1.54	1.44	3.04∗∗∗

*Note*: Each column reports coefficients and standard errors from an OLS regression of treatment status on all baseline characteristics. Test of joint significance reports F-stats on the null that all coefficients are jointly equal to zero. Standard errors clustered at the farmer-group-level are in parentheses (*∗∗∗*p<0.01*, ∗∗*p<0.05*, ∗*p<0.10).

### 3.3 Attrition

Our experimental design involved a baseline survey prior to randomization, random assigned prior to planting, and an endline survey seven months later, after harvest. Because of this time delay we did experience attrition among the farmers in our experiment. Of the 953 farmers interviewed at baseline, 98 farmers dropped out, an attrition rate of ten percent. To test for the presence of attrition bias, we compare outcome variables and covariates at baseline across the returning and attriting farmers. We also check for systematic differences between attritors and returners within each treatment arm.^16^

As in our balance check, we regress each variable on an indicator for if the farmer was an attritor. Columns 1 and 2 of Table 4 present means and standard deviations for attriting and returning farmers. The following six columns present coefficients and standard errors, clustered at the farmer-group-level, from OLS regressions. For example, the third column displays coefficients and standard errors on an indicator equal to one if the farmer attrited for the sub-population of farmers randomized into T1 or the pure control. We find that attriting farmers had significantly lower income per capita prior to the experiment than returning farmers. Attritors also tended to be older and less educated, suggesting that they may be less adept at farming than returning individuals. However, significantly more attritors reported that farming was their primary activity. To ensure our results are not due to attrition bias, we calculate bounds (Lee, 2009) on our primary outcomes.

**Table 4:** Baseline differences between attrited and returning farmers.

	Returning (n ​= ​855)	Attrited (n ​= ​98)	Differences between attrited and returning farmers within groups						
			[T-C]	[T1-C]	[T2-C]	[T3-C]	[T2-T1]	[T3-T1]	[T3-T2]
Rice area (ha)	0.812 (1.847)	0.723 (0.767)	−0.089 (0.107)	−0.082 (0.072)	−0.007 (0.158)	0.007 (0.191)	−0.219 (0.153)	−0.089 (0.127)	−0.140 (0.153)
Yield (kg/ha)	916.4 (1573)	599.1 (821.0)	−317.3∗∗ (122.7)	−180.5 (186.7)	−312.1 (201.6)	−324.5 (183.9)	−344.2 (179.6)	−309.3 (177.1)	−388.5∗ (161.5)
Market participation (%)	38.31 (41.12)	38.14 (42.41)	−0.162 (5.141)	5.511 (6.431)	2.417 (9.951)	2.204 (9.044)	−2.403 (6.170)	−1.783 (6.414)	−5.099 (6.854)
Income per capita (US$)	230.9 (353.4)	147.4 (224.9)	−83.49∗∗ (29.49)	−103.8∗ (41.05)	−72.34 (60.94)	−81.77∗ (35.48)	−88.69 (48.80)	−87.37∗ (36.62)	−73.44 (38.34)
Household size (n)	8.270 (3.897)	8.388 (4.060)	0.118 (0.548)	−0.847 (0.738)	0.664 (1.018)	−0.186 (0.929)	0.449 (0.678)	−0.021 (0.674)	0.519 (0.664)
Age of household head (years)	40.80 (10.08)	44.44 (11.08)	3.635∗∗ (1.215)	−0.228 (1.620)	6.252∗∗ (2.311)	4.254∗∗ (1.542)	2.831 (1.791)	2.468 (1.341)	5.095∗∗∗ (1.454)
Male headed household (=1)	0.560 (0.497)	0.571 (0.497)	0.011 (0.059)	−0.053 (0.088)	0.118 (0.092)	−0.045 (0.109)	0.063 (0.069)	−0.075 (0.073)	0.043 (0.075)
Experience producing rice (years)	8.533 (5.047)	9.378 (5.979)	0.844 (0.667)	−1.268 (1.070)	1.710 (1.363)	1.877∗ (0.937)	−0.144 (0.842)	0.742 (0.877)	1.491∗ (0.736)
Primary education (=1)	0.108 (0.310)	0.051 (0.221)	−0.057∗ (0.025)	−0.111∗∗∗ (0.016)	0.005 (0.060)	−0.077∗∗ (0.025)	−0.046 (0.039)	−0.082∗∗∗ (0.020)	−0.036 (0.033)
Farming is main activity (=1)	0.920 (0.271)	0.969 (0.173)	0.049∗∗ (0.017)	0.075∗∗∗ (0.018)	0.052 (0.030)	0.031 (0.030)	0.059∗ (0.026)	0.046∗ (0.021)	0.041 (0.023)
Training in rice production (=1)	0.519 (0.500)	0.429 (0.497)	−0.091 (0.062)	−0.181∗∗ (0.070)	0.023 (0.116)	−0.128 (0.115)	−0.054 (0.069)	−0.101 (0.081)	−0.088 (0.083)
Member of farm assoc. (=1)	0.966 (0.181)	0.959 (0.199)	−0.007 (0.023)	−0.064 (0.061)	0.027∗∗ (0.010)	0.001 (0.027)	−0.007 (0.034)	−0.019 (0.034)	0.009 (0.017)

*Note*: The first two columns report means of the data at baseline with standard deviations in parentheses. The final seven columns report coefficients and standard errors from OLS regressions of the variables of interest or the covariates on attrition status within different groups. Standard errors clustered at the farmer-group-level are in parentheses (*∗∗∗*p<0.01*, ∗∗*p<0.05*, ∗*p<0.10).

## 4 Empirical framework

### 4.1 Expected outcomes

We focus on estimating the direct impacts of randomly assigned farming contracts on four measures of rural transformation: rice area (ha), yields (kg/ha), market participation (%), and income per capita (US$). To estimate these impacts, we compare outcomes for treated farmers with the outcomes in the absence of the treatment. We are not only interested in the effect of being offered a farming contract but the effects of each contract characteristic. As such, we present a large complement of results comparing treatment (a contract) to control, comparing each contract to control, and comparing differences in outcomes between the various treatment groups.

We expect any contract that reduces price risk, increases technical efficiency, or eases capital constraints to positively and significantly affect all four outcome variables. When it comes to expected differences between the impacts of each contract, the effect size will be heterogeneous, depending on where the largest gains are to be had for each individual farmer. That said, *a priori* we expect contracts that address more of the limitations facing farmers to have larger impacts. Because of this, our prior is that T3, which combines a fixed-price, production-management, and input-supply contract, will result in larger and more significant impacts compared to either of the other two treatments. Similarly, T2, which includes the price guarantee (fixed-price) and the extension training (production-management), should have larger impacts than T1, which only includes the price guarantee.

### 4.2 Treatment effects

Because we have both baseline and endline data, we can estimate treatment effects using two different approaches. We first estimate the treatment effect using a simple OLS model:

(1) $$y_{ir}=\alpha +\delta_{OLS}T_{ir}+X_{ir}\beta +\rho_{r}+\varepsilon_{ir}$$

where $y_{ir}$ is the outcome of interest for farmer i in arrondissement (region) r. Let $T_{ir}$ be our indicator of treatment, variously defined, for the farmer and $\delta_{OLS}$ the coefficient on the OLS estimate of the treatment effect. In some specifications we include a vector of farm household characteristics, $X_{ir}$. Since our sampling was stratified at the arrondissement, we include arrondissement fixed effects, $\rho_{r}$, in all regressions. Lastly, $\varepsilon_{ir}$, is an idiosyncratic error term orthogonal to $T_{ir}$ as a result of our randomization. Because the treatment is randomized at the farmer group level, we cluster all standard errors at that level.

Our second estimator is an Analysis of Covariance (ANCOVA) estimate of the treatment effect:

(2) $$y_{ir}=\alpha +\delta_{ANCOVA}T_{ir}+\mu y_{ir,PRE}+X_{ir}\beta +\rho_{r}+\varepsilon_{ir}$$

Here $y_{ir,PRE}$ is the value of the outcome variable from the pre-treatment 2015-16 growing season and ${\text{δ}}_{\text{ANCOVA}}$ is the coefficient on the ANCOVA estimate of the treatment effect. The ANCOVA estimator has more power than the typical difference-in-difference estimator when autocorrelation is low (McKenzie, 2012), which it is in our sample.^17^ Again, standard errors are clustered at the unit of randomization.

### 4.3 Multiple hypothesis testing

Because we are testing a large number of hypotheses, it is possible that significant results emerge from our analysis due not to actual treatment effects but rather to chance. While the problems arising from multiple inference are well known, dating back to Bonferroni (1935), the literature has yet to arrive at a consensus on the best way to correct for multiple hypothesis testing. Some suggest adjusting only when making inferences for multiple outcomes (Anderson, 2008; Casey et al., 2012; Heckman et al., 2011; Kling et al., 2007) while others suggest correcting only for multiple subgroups (Lee and Shaikh, 2014). Still others suggest correcting for both multiple outcomes and subgroups (Heckman et al., 2010). Both Bonferroni (1935) and Holm (1979) have proposed their own ways to adjust *p*-values to correct for the familywise error rate (FWER), the probability of making at least one false discovery among a family of comparisons.. More recently, List et al. (2019) have developed a step-wise FWER testing procedure. Alternatively, Anderson (2008) and Ksoll et al. (2016) use sharpened *q*-values to adjust for the false discovery rate (FDR), the probability of making at least one false discovery among the discoveries already made. We take a catholic approach and present results, in Appendix B, from the Bonferroni adjustment, the Holm adjustment, List’s step-wise correction, and Anderson’s sharpened *q*-values.

## 5 Primary results

### 5.1 Impact of contract farming

Table 5 presents the treatment effects of a farmer being randomly assigned a production contract on four measures of rural transformation. We present results from OLS and ANCOVA regressions, without and with covariates. Panel A presents treatment effects on rice area, measured in hectares; Panel B presents treatment effects on yields, measured as kg of paddy rice harvest per hectare; Panel C presents treatment effects on market participation, measured as the percentage of harvested rice sold into the market; and Panel D presents treatment effects on income per capita, measured as the total value of farm and non-farm income in U.S. dollars divided by household size.

**Table 5:** Treatment effects of farming contract [T-C].

	OLS	OLS	ANCOVA	ANCOVA
	(1)	(2)	(3)	(4)
Panel A: rice area (ha)				
Treatment effect	0.199∗∗∗ (0.055)	0.179∗∗∗ (0.057)	0.199∗∗∗ (0.055)	0.179∗∗∗ (0.057)
Mean dependent variable in control	0.772			
Observations	855	855	855	855
R-squared	0.064	0.070	0.064	0.070
Arrondissement FE	Yes	Yes	Yes	Yes
Household covariates	No	Yes	No	Yes

Panel B: yield (kg/ha)				
Treatment effect	466.9∗∗∗ (98.08)	480.4∗∗∗ (105.9)	459.0∗∗∗ (98.03)	472.7∗∗∗ (105.5)
Mean dependent variable in control	1652			
Observations	855	855	855	855
R-squared	0.086	0.095	0.089	0.097
Arrondissement FE	Yes	Yes	Yes	Yes
Household covariates	No	Yes	No	Yes

Panel C: market participation (%)				
Treatment effect	32.95∗∗∗ (2.634)	34.80∗∗∗ (2.427)	32.97∗∗∗ (2.664)	34.85∗∗∗ (2.432)
Mean dependent variable in control	24.96			
Observations	855	855	855	855
R-squared	0.487	0.498	0.487	0.498
Arrondissement FE	Yes	Yes	Yes	Yes
Household covariates	No	Yes	No	Yes

Panel D: income per capita (US$)				
Treatment effect	120.0∗ (66.51)	120.3∗ (68.82)	158.5∗∗ (66.86)	138.9∗∗ (68.07)
Mean dependent variable in control	265.3			
Observations	855	855	855	855
R-squared	0.090	0.285	0.168	0.308
Arrondissement FE	Yes	Yes	Yes	Yes
Household covariates	No	Yes	No	Yes

*Note*: For simplicity, coefficient estimates are only reported for the treatment effect. Covariates include household size, age and gender of household head, number of years growing rice, and indicators for if the household head had at least primary education, if farming is the household’s main activity, if they have received extension training previously, and if they are a member of a farmer association. Standard errors clustered at the farmer-group-level are in parentheses (*∗∗∗*p<0.01*, ∗∗*p<0.05*, ∗*p<0.10).

Farmers randomly selected to receive a farm contract were provided with the written and signed contract prior to planting, which gave them time to reallocate their own land or bring in more land if they desired. Both the OLS and ANCOVA estimates reveal that farmers with a contract did plant a significant amount of additional land with rice compared to control farmers without a contract. Despite land being a lumpy input, farmers with contracts planted 23 percent more land with rice than control farmers, a half standard deviation shift above the control mean. Anecdotal evidence from the study reveals that farmers planted rice in lowland areas, so as to minimize the cost of irrigation. These lowland areas are typically used to cultivate home vegetable gardens or are left fallow. Farmers appear not to have substituted away from maize and cotton, the region’s primary crops, in order to plant rice but rather brought new marginal land into cultivation.

Examining results of farming contracts on the other three variables of interest, we also find consistently positive and significant effects. Focusing on the ANCOVA estimates with covariates, being offered a farming contract increases yields by 473 ​kg per hectare, a 29 percent increase in yields compared to the control. Given that we offered three types of contracts, this result does not immediately reveal what contract attributes most contributed to the yield gains. What is clear is that farmers did not simply fulfill their contracts by increasing the amount of land planted to rice. Rather, their productivity per unit of land increased in response to signing a farming contract.

Not unexpectedly, farmers with production contracts increase their market participation by selling 35 percentage points more of their rice harvest, a 140 percent increase above farmers without contracts. This result may appear tautological, as farmers with contracts are expected to sell the contracted quantity to ESOP. However, even with contracts, farmers sell well less than 100 percent of their rice crop, implying that farmers produce enough rice to meet the terms of their contract and are able to decide how to dispose of the excess quantity, either by consuming the rice or saving it as seed for next year.^18^

One concern in the existing literature on contract farming is that by signing a contract, farmers reallocate land and labor to the contracted crop. Thus, while farmers may increase their production on one crop, the overall income effect may be zero or negative (Bellemare, 2018; Ragasa et al., 2018). We find that farmers in the treatment earned $140 more per person, an increase of 52 percent or about four tenths of a standard deviation above the mean for control households. This is a substantial income gain in a country where GDP per capita is around $800. In considering how farmers increased yields and income, Table B3 in the Appendix presents results from ANCOVA estimates of treatment on seed, fertilizer, pesticide, herbicide, and labor. Treatment significantly increases the use of each input, indicating that the contracts resulted in an intensification of rice cultivation, in addition to the extensification show in the regressions of rice area on treatment.^19^

Overall, our results, the first from an RCT, provide consistent evidence that contract farming has a positive and significant impact on several measures of farm productivity and household welfare. At least for rice growing households in Benin, contract farming appears to be a mechanism that encourages vertical coordination and can contribute to rural transformation.

### 5.2 Impact of contract attributes

**Fig. 3:**
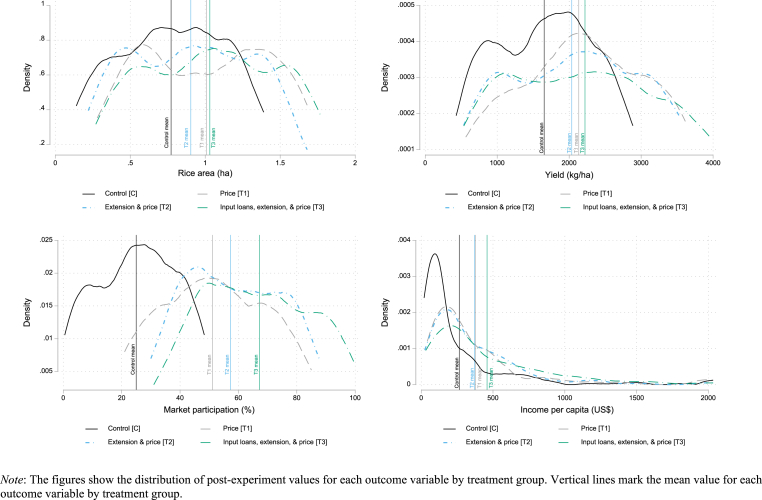
Outcomes by treatment group.

Given these positive results, it is particularly important to understand which contract attributes matter most in increasing yield and income. To do this, we randomly assigned treated households into one of three contract types. Fig. 3 summarizes the effect of each of the three types of contracts by drawing distributions of post-experiment values for each outcome. To the distributions we add vertical lines to mark the unconditional mean for each outcome variable by contract type. Visual inspection shows some heterogeneity in outcomes based on contract attributes. We also present regression analysis of these treatment effects, which not only allows us to test for differences between each treatment and the control but also test for differences between one treatment and another. Results from these regressions are presented in Table 6, with Bonferroni-adjusted Wald tests for differences between coefficients on the treatment dummies in Table 7.

**Table 6:** Treatment effects of each contract characteristic [T3-T2-T1-C].

	OLS	OLS	ANCOVA	ANCOVA
	(1)	(2)	(3)	(4)
Panel A: rice area (ha)				
Treatment effect of T1	0.247∗∗∗ (0.068)	0.228∗∗∗ (0.068)	0.247∗∗∗ (0.069)	0.228∗∗∗ (0.069)
Treatment effect of T2	0.134∗∗∗ (0.048)	0.115∗∗ (0.050)	0.133∗∗∗ (0.048)	0.114∗∗ (0.051)
Treatment effect of T3	0.268∗∗∗ (0.064)	0.251∗∗∗ (0.066)	0.268∗∗∗ (0.064)	0.251∗∗∗ (0.067)
Mean dependent variable in control	0.772			
Observations	855	855	855	855
R-squared	0.079	0.085	0.080	0.085
Arrondissement FE	Yes	Yes	Yes	Yes
Household covariates	No	Yes	No	Yes

Panel B: yield (kg/ha)				
Treatment effect of T1	520.4∗∗∗ (127.6)	518.8∗∗∗ (147.6)	508.9∗∗∗ (128.3)	506.6∗∗∗ (147.6)
Treatment effect of T2	424.9∗∗∗ (102.3)	453.5∗∗∗ (109.4)	418.8∗∗∗ (102.2)	447.6∗∗∗ (109.2)
Treatment effect of T3	502.8∗∗∗ (111.3)	506.5∗∗∗ (119.6)	494.5∗∗∗ (110.3)	497.8∗∗∗ (117.9)
Mean dependent variable in control	1652			
Observations	855	855	855	855
R-squared	0.088	0.096	0.090	0.098
Arrondissement FE	Yes	Yes	Yes	Yes
Household covariates	No	Yes	No	Yes

Panel C: market participation (%)				
Treatment effect of T1	22.11∗∗∗ (2.748)	23.87∗∗∗ (2.530)	22.12∗∗∗ (2.739)	23.89∗∗∗ (2.509)
Treatment effect of T2	31.43∗∗∗ (2.294)	32.28∗∗∗ (2.184)	31.43∗∗∗ (2.298)	32.29∗∗∗ (2.185)
Treatment effect of T3	39.05∗∗∗ (2.364)	40.76∗∗∗ (2.128)	39.05∗∗∗ (2.365)	40.77∗∗∗ (2.121)
Mean dependent variable in control	24.96			
Observations	855	855	855	855
R-squared	0.530	0.542	0.530	0.542
Arrondissement FE	Yes	Yes	Yes	Yes
Household covariates	No	Yes	No	Yes

Panel D: income per capita (US$)				
Treatment effect of T1	128.6∗ (69.98)	92.63 (72.76)	146.4∗∗ (69.61)	105.3 (71.87)
Treatment effect of T2	79.69 (73.42)	90.49 (74.70)	123.4 (75.62)	109.2 (74.83)
Treatment effect of T3	170.7∗∗ (68.78)	166.3∗∗ (68.63)	210.0∗∗∗ (67.24)	186.9∗∗∗ (67.51)
Mean dependent variable in control	265.3			
Observations	855	855	855	855
R-squared	0.097	0.291	0.175	0.314
Arrondissement FE	Yes	Yes	Yes	Yes
Household covariates	No	Yes	No	Yes

*Note*: For simplicity, coefficient estimates are only reported for the treatment effect. Covariates include household size, age and gender of household head, number of years growing rice, and indicators for if the household head had at least primary education, if farming is the household’s main activity, if they have received extension training previously, and if they are a member of a farmer association. Standard errors clustered at the farmer-group-level are in parentheses (*∗∗∗*p<0.01*, ∗∗*p<0.05*, ∗*p<0.10).

**Table 7:** Wald tests for differences between coefficients.

	OLS	OLS	ANCOVA	ANCOVA
	(1)	(2)	(3)	(4)
Panel A: rice area (ha)				
Difference btw T2 & T1	0.1038	0.1091	0.1032	0.1062
Difference btw T3 & T2	0.0031∗∗∗	0.0032∗∗∗	0.0026∗∗∗	0.0027∗∗∗
Difference btw T3 & T1	1.000	1.000	1.0000	1.0000
All pairwise comparisons	0.0029∗∗∗	0.0036∗∗∗	0.0026∗∗∗	0.0031∗∗∗
Panel B: yield (kg/ha)				
Difference btw T2 & T1	0.9821	1.0000	1.0000	1.0000
Difference btw T3 & T2	1.0000	1.0000	1.0000	1.0000
Difference btw T3 & T1	1.0000	1.0000	1.0000	1.0000
All pairwise comparisons	0.5109	0.7503	0.5310	0.7759
Panel C: market participation (%)				
Difference btw T2 & T1	0.0000∗∗∗	0.0001∗∗∗	0.0000∗∗∗	0.0001∗∗∗
Difference btw T3 & T2	0.0000∗∗∗	0.0000∗∗∗	0.0000∗∗∗	0.0000∗∗∗
Difference btw T3 & T1	0.0000∗∗∗	0.0000∗∗∗	0.0000∗∗∗	0.0000∗∗∗
All pairwise comparisons	0.0000∗∗∗	0.0000∗∗∗	0.0000∗∗∗	0.0000∗∗∗
Panel D: income per capita (US$)				
Difference btw T2 & T1	0.8798	1.0000	1.0000	1.0000
Difference btw T3 & T2	0.1661	0.1364	0.1799	0.1372
Difference btw T3 & T1	1.0000	0.2490	0.5347	0.1581
All pairwise comparisons	0.1541	0.0956∗	0.1596	0.0817∗

*Note*: Each cell contains the Bonferroni-adjusted *p*-values for Wald tests between coefficient estimates reported in Table 6. Significance of the test is reported as *∗∗∗*p<0.01*, ∗∗*p<0.05*, ∗*p<0.10.

All three contracts result in farmers increasing rice area relative to control farmers. However, testing for differences between the magnitudes of the coefficients reveals that the effect of T1 (fixed-price) is not significantly different from the effect of T2 (production-management) or T3 (input-supply). By comparison, the effect of T2 (production-management) on area planted to rice is significantly lower than the effect of T3. While one could expect that the provision of input loans lowers the per unit cost of production, allowing farmers to expand area planted to rice without increasing their total farm production costs, it is less obvious why farmers with a contract that only guaranteed a price planted a similar sized area. It may be that farmers who were to receive the extension services (T2) decided to focus effort on applying their training to a more circumscribed area. For those in T3, the addition of the input loan to the training may have reduced costs enough for farmers in this group to increase their area planted to an amount similar to those in T1. However, we lack the detailed farm production data needed to test this hypothesis.

Turning to each contract’s effect on yield, we find that all three have a positive and significant impact. The magnitude of the impact varies slightly, from about 450 ​kg per hectare for farmers in T2 to about 500 ​kg per hectare for farmers in T1 and T3. A Wald test for differences between each of these coefficients fails to reject the null of equality (Table 7). One possible explanation for this results is that, given the variance in yields, we lack power to detect significant differences across treatment arms. A second possible explanation is that farmers gained little in terms of productivity by receiving extension training or input loans. Table B4 in the Appendix provides ANCOVA estimates of each contract on input use. Each contract significantly increases seed, fertilizer, and labor use, though contracts tend to have a null effect on pesticide and herbicide use. Bonferroni-adjusted Wald tests for differences between coefficients on each contract indicator are never significant, indicating that across treatments farmers used about the same level of inputs. While far from conclusive, we take this as suggestive evidence that simply resolving price risk was sufficient to allow farmers to increase their use of inputs and thereby substantially increase yield.

All three contracts have a positive and significant impact on market participation. However, unlike yield, in which each contract’s effect size was statistically similar, the impact of each contract on market participation significantly differs from each other. Conforming with our priors, effect sizes are greater for contracts that offer more services to the farmer. Those in the T1 treatment sell just under 50 percent of their rice harvest into the market (24 percentage points more than the control), while those in T2 sell 57 percent and those in T3 sell 66 percent. The effects of using contracts to integrate farmers into the market are clear. Without a contract to produce rice, households sell about a quarter of their rice production and keep the remaining three quarters. Under the most complex contract, farmers nearly reverse this ratio, selling almost 70 percent of their rice into the market and retaining only 30 percent.

The evidence for each type of contract’s impact on income per capita is less obvious than when we simply compare all contracts to no contract, as in Table 5. While the effects of all three contracts are positive relative to the control, only the effect of the input-supply contract (T3) is consistently significant. Yet, when we conduct Wald tests for differences between coefficients, we consistently fail to reject the null of equality. We speculate that this is due to a lack of power sufficient to detect differences in effect size in a notoriously noisy variable such as income and not evidence of a true null. We base this on the similarity in the size of coefficients and standard errors across the income regressions in Table 5, Table 6.

Overall, we find a curious degree of variation in impacts based on the terms of the contract. Contrary to our priors, it is not always the case that the effect size of T1 is smaller than T2, which is smaller than T3. Instead, we find that the fixed-price contract (T1) increases rice area to the same extent as the input-supply contract (T3), while the production-management contract (T2) has a smaller effect. All three contracts have similar effects on yields, meaning that the provisioning of extension training and/or input loans does not result in increased yield relative to the contract the only provides a price guarantee. For income per capita we again find that the added elements of T2 and T3 do not seem to provide much additional value over the simple fixed-price contract. Throughout the analysis, we frequently find that the magnitude of the coefficient on the T2 treatment is the smallest of the three treatment arms, while the magnitude of the coefficient on the T1 treatment is only slightly less than that on the T3 treatment. In fact, the only outcome variable that conforms to our prior is market participation, where farmers with the production-management contract sell significantly more rice than farmers with the fixed-price contract, and farmers with the input-supply contract sell significantly more rice than the other two.

## 6 Heterogeneity analysis and robustness checks

### 6.1 Heterogeneity

A consistent piece of evidence from non-experimental studies on contract farming is that the benefits of participation are not uniform across farmer type. Bellemare (2012) finds that older and more educated households reap more of the rewards from contracting across a number of different crops in Madagascar. Michelson (2013) shows that the majority of benefits from vegetable contracts in Nicaragua accrue to those living along major roads. Narayanan (2014) finds that in Southern India income varies depending on which crop farmers choose to grow. Cahyadi and Waibel (2016) find that the positive impacts of oil palm contracts in Indonesia are contained to the relatively wealthy. A number of studies find that gender plays a role not just in participation in contract farming but in circumscribing the benefits of participation (Wang et al., 2014a,b; Bellemare and Bloem, 2018).

We investigate the presence of heterogeneity in our experimental data by estimating a regression in which the treatment dummy is interacted with various baseline characteristics:

(3) $$y_{ir}=\alpha +\delta_{ANCOVA}T_{ir}+\delta_{Z}Z_{i}+\delta_{H}\left(T_{ir}\times Z_{ir}\right)+\mu y_{ir,PRE}+X_{ir}\beta +\rho_{r}+\varepsilon_{ir}.$$

Here, $Z_{i}$ is the baseline characteristic of interest and all other variables are as defined for the ANCOVA regression with covariates. Our coefficient of interest is ${\text{δ}}_{\text{H}}$ and we focus our analysis on the covariates presented in Table 2.^20^

Table 8 presents the results of our heterogeneity analysis by baseline covariate. Columns display ANCOVA results for the four outcome variables as the dependent variable. Each row designates which covariate is interacted with the treatment indicator. Cells report the coefficient and standard error on the interaction term of household covariate (row) and treatment indicator on the dependent variable (column). We find almost no evidence of heterogeneous treatment effects by baseline characteristics. We fail to reject the null that any of the covariates mitigate or accentuate the effect of contract farming on the area of land put into rice production. For yields and market participation, a marginally significant degree of heterogeneity exists based on a farmer’s previous training in rice production. For income per capita, the only interactions that are significant are household size with the contract and experience producing rice with the contract. In both cases, larger households and more experienced rice producers had lower income with the contract than similar households without the contract.

**Table 8:** Heterogeneity of farming contract treatment effects [T-C].

	Rice Area (ha)	Yield (kg/ha)	Market Participation (%)	Income per Capita (US$)
	(1)	(2)	(3)	(4)
Household size (n)	−0.003 (0.009)	−2.919 (16.86)	−0.175 (0.337)	−12.18∗ (7.216)
Age of household head (years)	0.003 (0.003)	2.018 (7.329)	−0.079 (0.156)	−2.682 (3.175)
Male headed household (=1)	0.081 (0.068)	−87.07 (130.2)	−2.953 (2.487)	45.55 (48.75)
Experience producing rice (years)	−0.009 (0.009)	2.643 (17.13)	−0.166 (0.261)	−13.97∗ (7.075)
Primary education (=1)	−0.098 (0.097)	214.1 (183.3)	2.091 (3.573)	6.050 (79.74)
Farming is main activity (=1)	0.031 (0.126)	283.0 (236.5)	2.175 (4.230)	169.5 (130.3)
Training in rice production (=1)	0.054 (0.072)	230.8∗ (138.5)	5.068∗ (2.599)	−24.43 (58.60)
Member of farm assoc. (=1)	0.121 (0.173)	300.1 (294.5)	−1.347 (9.146)	−117.1 (96.99)

*Note*: Columns present ANCOVA regressions with covariates and arrondissement fixed effects for the four outcome variables as the dependent variable. Each row designates which covariate is interacted with the treatment indicator. Cells report the coefficient and standard error on the interaction term of covariate (row) and treatment indicator on the dependent variable (column). Standard errors clustered at the farmer-group-level are in parentheses (*∗∗∗*p<0.01*, ∗∗*p<0.05*, ∗*p<0.10).

**Fig. 4:**
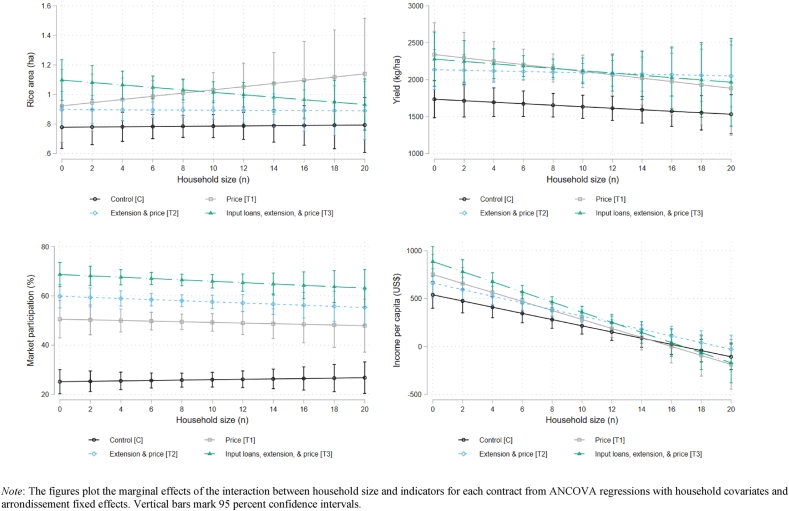
Heterogeneous effects of household size.

To provide a more detailed exploration regarding these three potential sources of heterogeneity, we graph the marginal effects of each interaction term on our outcome variables. Fig. 4 plots the marginal effects and 95 percent confidence intervals for the interactions between household size and indicators for each type of production contract. Panels document the effect on one of the four outcome variables. As was evident from Table 8, there is a lack of heterogeneity in household size on rice area, yield, and market participation. For income per capita, we find that smaller households offered the input-supply contract (T3) have higher income than control households of similarly small size. As household size increases, income per capita for all groups decreases until there are no significant differences across treatments. It appears that when households are relatively small (1–8 people), they are better able to take advantage of being offered the input-supply contract and convert it into more income for each member. This difference diminishes for households with more than eight members and is not significant for the other treatment arms.

**Fig. 5:**
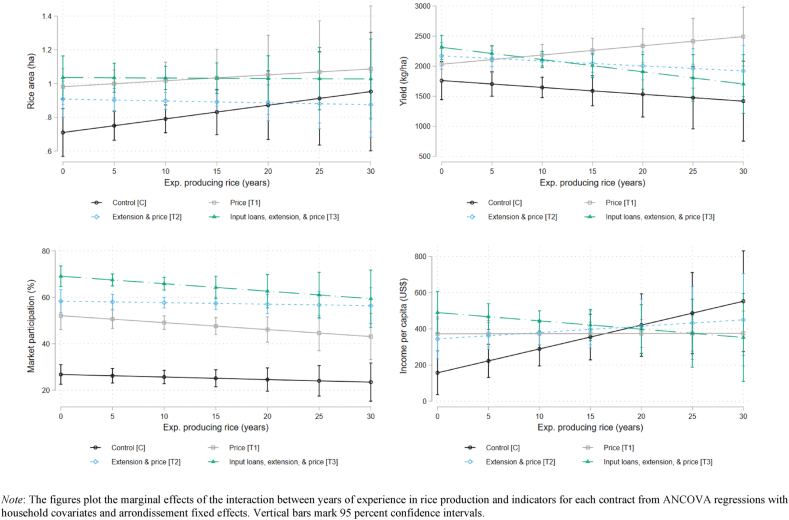
Heterogeneous effects of experience producing rice.

Fig. 5 presents a similar set of margin plots for the effect of experience in rice production, measured in years. As with household size, there is little evidence of heterogeneous treatment effects on rice area, yield, or market participation. Where significant evidence does exist is for income per capita. Without a contract, more experienced farmers have higher income than less experienced farmers. This heterogeneity based on experience disappears for farmers randomly assigned to a production contract. Regardless of the type of contract, less experienced farmers have approximately the same amount of income as more experienced farmers. Contract farming helps inexperienced farmers earn incomes comparable to that earned by much more experienced farmers. It takes farmers without a contract a decade or more of experience to earn similar levels of income.

**Fig. 6:**
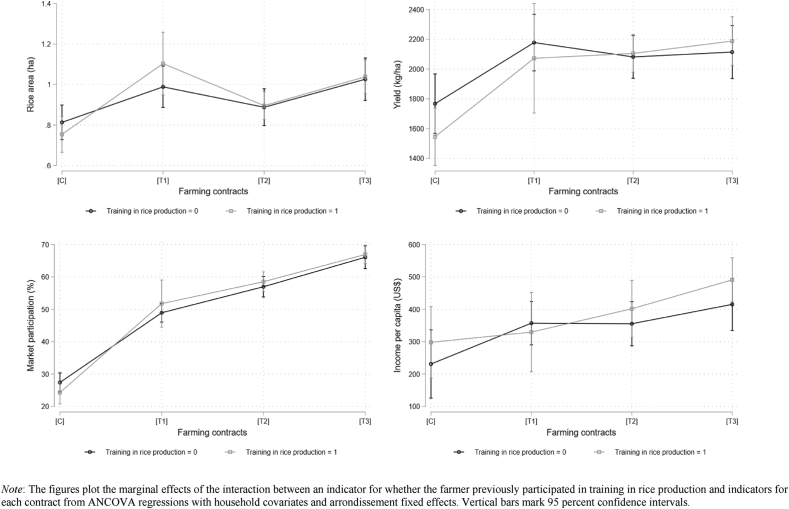
Heterogeneous effects of participated in training in rice production.

Finally, Fig. 6 graphs the marginal effects of each treatment interacted with an indicator for whether or not the farmer had participated in training in the last 12 months. Because the household characteristic is now a binary variable, we graph each contract along the horizontal axis and the lines represent if the farmer participated in training. Here again we find little evidence of heterogeneity. As was evident in Table 8, households with training and a contract had higher yields and greater market participation than households with training in the control. But there are no significant differences across treatment arms and no significant differences within treatment arms across training/no-training.

To some extent, our heterogeneity analysis appears fruitless. Across a number of different pre-specified covariates and a number of different outcome variables, we fail to find much evidence of heterogeneity in treatment effects. Even when we disaggregate the contract treatment by contract attributes, there is little evidence of significant differences. Yet, the plethora of null results arising from the heterogeneity analysis is informative regarding both the contracts offered to participants in the experiment and the larger non-experimental literature as a whole. The contracts offered by ESOP appear to benefit households equally. The contracts did not disadvantage female headed households or older farmers. Nor did they advantage farmers with more experience or those who participated in training programs or were members of local farm associations. This uniformity is encouraging regarding the equity and achievability of the contracting terms that ESOP offers. In regards to the larger literature on the heterogeneous effects of contract farming, endogenous matching between principals and agents is extremely difficult to control in non-experimental settings (Ackerberg and Botticini, 2002). It may be the case that some non-experimental studies have confounded heterogeneity in contract outcomes with heterogeneity in contract participation.

### 6.2 Robustness

The final component of our analysis is to explore the robustness of our primary results to different samples, different inference, and different specifications. Table 9 summarizes these robustness checks and their results. The table also points to where in the Appendix the complete results for each check can be found.

**Table 9:** Summary of robustness checks.

Robustness check	Result
Account for attrition with Lee (2009) bounds	Estimates are bounded away from zero (see Table B5).
Account for multiple hypothesis testing	
With Bonferroni (1935) adjustment	No change in significance (see Table B6 and Table B8).
With Holm (1979) adjustment	No change in significance (see Table B6 and Table B8).
With List et al. (2019) adjustment	No change in significance (see Table B6 and Table B8).
With Anderson (2008) sharpened *q*-values	No change in significance (see Table B6 and Table B8).
Randomization inference using Heβ (2017)	Loss of significance for the impact of T1 on rice area. Significance of remaining treatments on outcomes not due to random chance (see Table B7 and Table B9).
Pairwise comparison of each treatment to control	Loss of significance for the impact of T1 on rice area and on income per capita in three specifications (see Table B10, Table B11, and Table B12).
Pairwise comparison between treatment arms	Increase in differences between treatment arms for several outcomes (see Table B13, Table B14, and Table B15).
Alternative measures of household welfare	Contracts increase rice income without reducing income from other sources (see Table B16 and Table B17). Contracts significantly increase Food Consumption Score and marginally decrease Household Food Insecurity Access Scale (see Table B18 and Table B19).

*Note*: Table summarizes the results from robustness checks. Full results are available in the Appendix.

Our first check is whether our inference is robust when we account for attrition. Since some differences do exist between attritors and non-attritors in our sample, we calculate bounds for our estimates following Lee (2009) and accounting for the stratified and clustered design of the experiment. All point estimates for the treatment effect are bounded away from zero (see Table B5 in the Appendix). These results suggest that any potential bias introduced by differences between attriting farmers and returning farmers is small relative to our estimated effect sizes.

Next, we check whether our inference is robust to corrections that account for testing multiple hypotheses. We adjust p-values for the FWER using the Bonferroni (1935), Holm (1979) and List et al. (2019) correction, along with adjusting for the FDR using Anderson (2008) sharpened *q*-values. Corrections for our main results are in Table B6 and Table B8 in the Appendix. In general, there is no change in significance from any of these corrections. In cases where our point estimates are significant, they remain significant when accounting for multiple hypothesis testing.

Given the grouped nature of the randomization, leaving us with 107 treatment units, it is possible that asymptotic inference is unreliable. As an alternative method for interrogating the robustness of our results, we implement a randomization inference procedure outlined in Heβ (2017). Where classical inference assumes the treatment is fixed and the sample is a random draw, randomization inference assumes the sample is fixed and the assignment to treatment is random. For each ANCOVA regression with covariates, we randomly permute the treatment indicator 5000 times, accounting for the stratified and clustered design of the experiment, which allows us to build a reference sample under the sharp null hypothesis of no treatment effect. We can then compare the distribution of outcomes when the hypothetical treatment effect is zero with the observed treatment effect and calculate *p*-values. Table B6 and Table B7 in the Appendix present *p*-values from the analytical standard errors presented in the body of the paper along with *p*-values calculated from the randomization inference procedure. Similar to our adjustments for attrition and multiple hypothesis testing, randomization inference does not move our results from significant to not significant for the pooled treatment. When it comes to the treatment effects for each contract type, there is a change in the effect of T1 on rice area, which is no longer significant. Besides this case, all other significant treatment effects remain significant, though at reduced levels.

Our next two checks are concerned with the robustness of our results to difference specifications. In Table 6 we presented results from regressions which included indicators for each treatment. We now conduct a pairwise comparison of each treatment against the control (see Tables B10–B12 in the Appendix). We find that several estimates of the effect of the fixed-price contract (T1) on rice area are no longer significant. Additionally, the OLS estimate without covariates of the effect of T1 on income per capita is no longer significant. There is no change in significance for any of the other variables and specifications. In Table 7 we presented Bonferroni-adjusted Wald tests comparing coefficient sizes across treatments. We can make this same comparison in a pairwise fashion, directly testing T2 against T1, T3 against T1, and T3 against T2 (see Tables B13–B15 in the Appendix). Any differences that were significant using the Wald test remain significant in the pairwise comparisons. However, we find that three cases where the Wald test failed to reject the null can be rejected in a pairwise comparison. Rice area for those in T2 is significantly less than T1 and income per capita is significantly larger for those in T3 compared to both T2 and T1. In each of these cases the p-value of the Wald test fell just below the 90 percent critical value and the switch to a direct comparison increases the precision of the estimates.

Our final robustness check is to test our results using alternative measures of welfare. First, we disaggregate income into rice income, farm income other than from rice, and non-farm income. This allows us to determine if households are reallocating effort away from other sources of income and towards rice production. Tables B16 and B17 in the Appendix present results for the pooled treatment and each treatment arm. The results are consistent across tables: the treatment increases rice income but does not have a significant effect on other income sources. However, there is a small but insignificant negative effect on non-rice farm income. Despite this, it appears that contract farming increases household income without reducing other sources of income.

Second, since income is notoriously difficult to measure accurately, we estimate treatment effects on two food security metrics. The first is the Household Food Insecurity Access Scale (HFIAS) and the second is the Food Consumption Score (FCS). The HFIAS measures a household’s feelings and perceptions of food insecurity and is the preferred measure of USAID. The FCS measures how often a household consumes food items in different food groups and is the preferred measure of the World Food Programme.^21^ Our results are generally robust to these alternative welfare measures (see Tables B18 and B19 in the Appendix). Farming contracts increase the FCS as do all three individual contracts. However, there is little evidence that farming contracts reduce the HFIAS. While some of the treatment effects on HFIAS are significant, they tend not to be robust to adjustments accounting for multiple hypothesis testing or attrition.

Third, we estimate treatment effects on a back-of-the-envelope calculation of profits from rice production.^22^ Our data contains detailed inputs on rice production, including labor time. However, it lacks detailed price data on hired and household wages as well as input data on total farm production. Despite these limitations in the data, and the long-standing problem of valuing family labor, we can compute a rough estimate of profits earned from rice production. We use three different wages to calculate a range of rice profits. Based on data from ESOP, we calculate profits at a “low wage rate” of 1500 CFA per day and a “high wage rate” of 2000 CFA per day. We also calculate profit using self-reported “cost of labor” for rice production. Using these three sources to value both hired and family labor, we can create a range of back-of-the-envelope calculations for rice profits per hectare. As can be seen in Tables B20 and B21 in the Appendix, farm contracts increase rice profits when self-reported labor is used but have no significant effect when we use the low and high wage data from ESOP. We believe that the lack of impact on profit using ESOP-reported wages is due to a lack of precision in calculated profit, and, as a result, a lack of precision in estimates. Using self-reported costs to calculate wage rates, standard errors on estimates are always below 1.0. However, standard errors on estimates using ESOP data are frequently above 2.0. We conclude that there is suggestive, though far from conclusive, evidence that the contracts did in fact increase profits.

## 7 Discussion

The results from our field experiment present consistent evidence regarding the impact of contract farming, though somewhat unexpected insights regarding the impact of different contract attributes. Participation in contract farming, or at least the contracts ESOP offered to rice farmers in our study, has a positive and significant impact on area planted, yield, market participation, and income. Obviously, this should not be interpreted as definitive evidence that all contract farming is beneficial to the agent, as contract terms will vary based on context, bargaining power, and the objective function of the principal.

While the overall positive effect of a farm contract was expected, we did not anticipate some of the differences in outcomes across contract type. In particular, contracts that provide extension training seemed to add no value above and beyond the fixed-price contract. Evidence from comparisons in Table 6 and our robustness checks all show that the provision of extension training frequently resulted in lower outcomes (though not always significantly lower) relative to the other contracts. Similarly, the estimates of treatment effects on input use does not reveal substantial differences between contracts that provided extension services and the contract that did not.

Three factors may explain these results. First, extension training is expected to increase technical efficiency. However, many smallholder farmers are resource-poor and may be unable to apply the knowledge they have gained. For instance, training regarding best practices for the application of fertilizer when the farmer cannot afford to buy the fertilizer is time ill spent. Second, the farmers in our experiment had very basic levels of education. The extension training developed with ESOP may have been pitched at too high a level to be effective. Third, it may be the case that the extension training was too broad. Recent RCT evidence from Kenya and Nigeria has shown that significant improvements can be made to agricultural outcomes when targeted or personalized advice is offered (Arouna et al., 2021; Tjernström et al., 2019). By comparison, broad or generalized recommendations typically provide no value added to farmers. That extension training was ineffective in our study is disappointing but not abnormal. Feder et al. (2010), Bellemare (2010) and Jones and Kondylis (2018) all provide evidence that extension services in developing countries often prove ineffective in producing positive and significant outcomes for smallholder farmers. Furthermore, in many developing countries, extension services focus more on cash crops (cotton, cocoa, peanut, palm oil, etc.), neglecting staple food crops such as rice (Diagne and Pesche, 1995).

While extension training proved to provide little added value, the simple fixed-price contracts turned out to produce particularly large impacts. Across multiple comparison groups, the fixed-price contract resulted in outcomes statistically indistinguishable from the contract that added input loans and extension training to the price guarantee. Focusing on the results of the Wald tests in Table 7, the contract that only offered a fixed price had similar effect sizes for area planted, yields, and income relative to the contract that added extension services and input loans. Market participation was the only outcome variable where the fixed-price contract failed to meet or exceed the effect size of one or more of the other contracts.

This result is striking in its simplicity and enormously encouraging in its implications for contract farming and rural transformation. It implies that the primary issue facing these farmers is output price risk. Though our experimental design does not allow for a clean test of the effect of eliminating price risk, since all contracts include non-price attributes, the preponderance of evidence suggests that providing a contract that eliminates price risk allows farmers to, on their own, make the necessarily investment to increase their rice area, increase their productivity, and, by selling more rice into the market, increase their income. Our results regarding the role of output price risk closely align with evidence presented in Michelson et al. (2012) and Michelson (2013) regarding contract farming schemes in Nicaragua. There the authors study contracts offered by Walmart and other supermarkets to purchase produce from smallholder farmers. They find that farmers who receive contracts isolating them from fluctuations in outprice take on more credit, farm more intensively, produce more, and earn a higher income.

Our results demonstrate experimentally what has long been argued anecdotally, that farmers respond to price incentives (Schultz, 1964). For organizations looking to provide contracts to farmers, this result is encouraging because it implies that they can provide strong incentives to farmers without undertaking the costs of providing training and input loans. By far the most binding constraint to expansion for ESOP is the need to raise sufficient capital to provide input loans to farmers at planting. Our results demonstrate that much of this expense may be unnecessary and ESOP could potentially expand the number of farmers it contracts with, and thus its throughput, by offering farmers a guaranteed price. With a price guarantee delivering secure market access, farmers can use the contract as collateral to rent in more land and obtain loans for inputs, improving outcomes for both parties and contributing to more rapid rural transformation.

## 8 Conclusion

The use of contract farming has a long tradition in modern agriculture and has been proposed as an engine for rural transformation, not just an outcome from the modernization of agriculture. However, concrete evidence for or against the role of contract farming in rural transformation has been lacking. Previous studies have been exclusively observational, and many studies have attempted to draw causal inference from cross-sectional data. Our study provides experimental evidence of the impacts of contract farming in a developing country context.

The results demonstrate that contract farming has positive and significant impacts on a number of different measures of farm productivity and household welfare. Of particular interest to both contracting parties as well as policymakers are the strong effects provided by a simple fixed-price contract. The provision of the fixed-price contract results in outcomes frequently indistinguishable from more complex (and costlier) contracts that provide extension training and/or input loans. This suggests that once price risk is resolved, farmers are able to, on their own, address issues of technical efficiency and capital constraints.

A caveat, as with any experimental study, is that the external validity of our results may be limited. Yet, we believe that our experiment provides a context and setting more generalizable than most observational studies of contract farming. Observational studies have frequently focused on high-value or specialty crops, cultivated by a small number of farmers relative to the number cultivating staple crops. In comparison, we study contract farming for a staple grain. Unlike specialty crops, the margins for staple crop cultivation are small. This suggests that our results should not only be generalizable to contract farming for other staple crops but may be a lower bound on the impacts that contract farming has on specialty crops, where more surplus exists.

Finally, the outcomes from our experiment support older theoretical and newer empirical research that conceptualize the persistence of the agrarian status quo as a mechanism design problem. In this perspective, the constraints to structural transformation are best understood using the tools of information economics, as opposed to broadly framing the issues as government or market failure. Interventions are less about political economy and more about addressing the presence of asymmetric information and moral hazard through mechanism design. We show that careful design of production contracts can allow for better vertical coordination in the agricultural sector, improve the productivity and income of farmers, and help foster the process of rural transformation.

## Declaration of competing interest

We, Aminou Arouna, Jeffrey D. Michler and Jourdain C. Lokossou the authors of the manuscript entitled “Contract Farming and Rural Transformation: Evidence from a Field Experiment in Benin”, declare that we have no relevant or material financial interests that relate to the research described in this paper.

## Data Availability

Replication data and code will be uploaded with submission if accepted.

## Footnotes

1 Studies have found positive impacts of contract farming on income (Bellemare, 2012; Gatto et al., 2017; Maertens and Vande Velde, 2017; Bellemare and Lim, 2018; Soullier and Moustier, 2018), on food security (Miyata et al., 2009; Bellemare and Novak, 2017; Bellemare and Lim, 2018; Mishra et al., 2018; Soullier and Moustier, 2018), on assets (Michelson, 2013), on subjective wellbeing (Vath et al., 2019), and on increased input use (Deb and Suri, 2013). The literature has documented problems affecting contract farming performance, which include biased terms of trade (Singh, 2002), below market prices (Michelson et al., 2012), higher production costs (Ragasa et al., 2018), lack of compensation for crop failure (Guo et al., 2005), and high opportunity costs (Bellemare, 2018).

2 de Janvry et al. (2017) detail four field experiments that involve contracts in agricultural settings. Among those discussed, the studies examine contracts for insurance, credit, or tenure, and not contracts for farm production. Ashraf et al. (2009) might be a potential candidate for the first field experiment on contract farming. However, the authors randomize “services offered” by an NGO that helps farmers export crops, not with the processing and export firm itself. While these services resemble what might be provided as part of a farming contract, Ashraf et al. never refer to the treatments as contracts.

3 This contract, as with all the contracts, also specifies a number of non-price attributes such as the quantity which the processor will purchase and the requisite level of quality. Detailed specifics of each contract is discussed in section 3.2 and English translations of the contracts can be found in Appendix A.

4 See Wang et al. (2014b), Otsuka et al. (2016), Bellemare and Bloem (2018), and Ton et al. (2018) for reviews of the empirical literature on contract farming and its challenges with identification.

5 Examples include catfish (Trifković, 2014), horticulture (Michelson, 2013; Narayanan, 2014; Wang et al., 2014a; Mishra et al., 2018), flowers (Macchiavello and Morjaria, 2015), palm oil (Cahyadi and Waibel, 2016; Gatto et al., 2017), and coffee (Blouin and Macchiavello, 2019).

6 Due to delays in the release of some funds, the survey team was unable to conduct the baseline immediately after harvest (January 2016).

7 While farmers were instructed to form groups of eight, in reality group size varied. Mean group size ended up being 8.9 farmers. In total, we had 953 farmers organized into 107 farmer-groups.

8 Side selling is frequently a concern in production contracts. We worked to proactively address potential side selling by discussing the issue with farmers when they were randomly assigned to each contract. It was also raised during the baseline and mid-line surveys. Finally, the visits by extension agents were designed to also encourage farmers to refrain from side-selling. Based on our endline survey, and conversations with ESOP, there was no evidence of side-selling.

9 The sale price is equivalent to US$0.27 ​at an average exchange rate of US$1 ​= ​550 CFA.

10 In our subsequent analysis, we focus on the fixed-price element of this contract, though it should be noted that the existence of clauses governing non-price features means the contract does not allow for a clean test of “fixed-price only” versus the pure control. However, a contract that failed to provide direction on quantity, quality, variety, or delivery would be an incomplete contract, making it highly susceptible to breach by either parties.

11 Based on anecdotal evidence, interest rates for agricultural loans in the area range between 10 and 19 percent.

12 An obvious concern is that the priced offered by ESOP has an income effect in addition to the eliminating price risk. Based on our endline data, farmers in the control group received, on average 130 CFA per kg of rice, which is 20 CFA less than those in the treatment. While treatment farmers clearly got a higher price, the difference amounts to just under US$0.04 per kg.

13 Paddy rice is not directly sold to consumers since it requires processing. Paddy is either parboiled and milled (parboiled rice) or milled without parboiling (white rice). Farmers can sell paddy directly to a processor (with or without a contract) or they can sell it into the paddy market, where dealers aggregate quantities for sale to a processor.

14 See Deutschmann et al. (2019) for an example of the (lack of) effect of GPS-recorded versus self-reported land area on yield in the context of an RCT.

15 Table B2 in the Appendix presents balance tests between the pooled treatment of “any contract” and the control.

16 Table B2 in the Appendix also presents tests for attrition bias between the pooled treatment of “any contract” and the control.

17 McKenzie (2012) defines “low” autocorrelation as below 0.40. The autocorrelation between each of our outcome variables is: rice area (0.02), yield (−0.03), market participation (0.12), and income per capita (0.32).

18 While contracts with ESOP define a certain level of quantity, this value is viewed as the minimum amount farmers are expected to deliver. Historically, ESOP’s rice mill has operated below capacity, which is why they have sought to secure throughput using farming contracts. ESOP’s willingness to buy rice in an amount above what was stipulated in the contract was made clear to farmers at contract signing. While theoretically it is possible for farmers to engage in arbitrage, purchasing rice in the market and selling it to ESOP, the high transportation costs for such a bulky commodity make the opportunity unprofitable.

19 While it would be ideal to measure treatment effects on profit, we lack the data necessary to calculate shadow values for family farm labor, and thus lack the ability to calculate profit.

20 Our heterogeneity analysis follows as closely as possible to that defined in our pre-analysis plan (Arouna and Michler, 2019). Due to data issues, we have deviated from the plan by not checking heterogeneity in historic (pre-2015) production rates, subjective discount rates, asset ownership, and prior contracting experience.

21 Both of these metrics are specified in our pre-analysis plan. Variable construction follows standard practice.

22 The following results on profits were not specified in our pre-analysis plan but were undertaken at the suggestion of an anonymous reviewer and the editor.

Appendix Supplementary data related to this article can be found at https://doi.org/10.1016/j.jdeveco.2021.102626.
