# Supplementary material for: Contract farming and rural transformation: Evidence from a field experiment in Benin
Source: J Dev Econ. 2021 Jun;151:102626. doi: 10.1016/j.jdeveco.2021.102626 (PMC8214072; doi:10.1016/j.jdeveco.2021.102626)
Supplement: Multimedia component 1 [file mmc1.docx]

Online Appendix for:

Contract Farming and Rural Transformation:

Evidence from a Field Experiment in Benin

Aminou Arouna, Jeffrey D. Michler, and Jourdain C. Lokossou

January 2021

The material contained herein is supplementary to the article named in the title and published in the *Journal of Development Economics* (*JDE*).

## **Appendix A: Translations of farming contracts**

The following are English translations of the three contracts ESOP offered, at random, to farmers in the study. The first contract only provides a price guarantee. The second contract combines the price guarantee with extension training. The third contract adds the provision of seed and fertilizer to the price guarantee and extension training.

Each contract contains 17 clauses, which can be grouped into three types of clauses. The first type are those clauses that are identical across all three types of contracts and for every farmer. These include Clause 1, which defines ESOP as the principle, and Clauses 2 and 10, which state that the contract is binding. Also included in this first type of clause are those that govern the variety of rice, its quality, and the delivery of harvest. Clause 6 stipulates that the farmer must grow rice variety IR841, a common variety easily available in the market. Clause 7 stipulates the percentage of impurities (rocks, husks, stalks, etc.) allowable, which was set at two percent. Clauses 12 and 14 stipulate the collection point for the rice (the farmer group) and how the rice is to be packages (100 kg bags). Most importantly, Clause 8 sets the price for rice per kg, which in our experiment was set at 150 CFA for all participants.

The second type of clause are those that vary by farmer. These include Clause 3, which stipulates how much rice the farmer plans to produce and sell, and Clause 9, which stipulates the date of delivery. Additional farmer specific clauses define the length of the contract (Clause 11 and 15), whether the cost of the 100 kg bags is recoverable (Clause 13), to whom the payment will be made (Clause 16), and who will adjudicate disputes (Clause 17).

The final type of clause are those that vary by treatment group. These include Clause 5, which controls the provision of extension services. For those with a fixed-price contract (T1), this clause states that no extension services will be provided. In the other two contracts, this clause states that ESOP will provide training and lists the topics of the training. The final clause is Clause 4, which controls the provision of inputs. For those with a fixed-price (T1) or production-management contract (T2), this clause states that no seed or fertilizer will be provided. For those with an input-supply contract (T3), this clause states that ESOP will provide seed and fertilizer on loan. Though a farmer can choose how much rice they plan to cultivate, ESOP provides the same ratio of inputs to every farmer in T3: 45 kg of seed and 150 kg of fertilizer for every one hectare cultivated. So, if a farmer chooses to cultivate a hectare, that farmer would get 45 kg of seed and 150 kg of fertilizer. If instead the farmer decided to cultivate two hectares, the farmer would get twice the amount of inputs.

Object of Contract: Production of rice paddy by ………………………………… for the delivery to …………………………………

**CONTRACT**

**Contract Partners**:

Partner 1: Last and First Names: …………………………………

Residence / Location …………………………………

Contact Number: …………………………………

Function: …………………………………

Partner 2: Last and First Names: …………………………………

Residence / Location: …………………………………

Contact Number: …………………………………

Function: …………………………………

Both parties agree to undertake (respect) the following clauses:

Clause 1: Partner 1 is the initiator of the present contract

Clause 2: Both parties must respect the contract

Clause 3: Partner 1 agrees to buy ……………kilograms of rice paddy produced by Partner 2

Clause 4: Partner 1 will not provide any input to the production by Partner 2

Clause 5: Partner 1 will not provide any technical or training assistance to Partner 2

Clause 6: Partner 2 commits to providing rice of the variety *IR841* to Partner 1

Clause 7: Partner 2 is committed to providing rice of ……… percent of impurities to Partner 1

Clause 8: Partner 2 agrees to sell paddy rice at …………..….FCFA/kilogram to Partner 1. Partner 1 agrees to buy rice paddy at …………..………….FCFA/kilogram

Clause 9: Partner 2 agrees to deliver the rice paddy in the month of ……….…………. in the year of ……………………..

Clause 10: Both partners commit to be faithful to their commitments.

Clause 11: The present contract will last …….......…..months from ...…/…../……

Clause 12: Delivery of the rice will be in the village group.

Clause 13: The packaging of rice paddy are lost or recoverable.

Clause 14: Paddy rice will be delivered in 100 kilogram bags for packaging of 80 kilogram.

Clause 15: the present contract is a contract: (fixed period/duration undetermined)

Clause 16: Payment for rice for Mr./Mrs. ……………… will……………… (*in kind* / in cash)

Clause 17: In case of conflict, the regulation will be in ………………………………… (friendly / court)

***Partner 1 Signature Partner 2 Signature***

***First and last name First and last name***

***Witnesses***

***First and last name First and last name***

***Made in …………………the …/……/201……..***

Object of Contract: Production of rice paddy by ………………………………… for the delivery to …………………………………

**CONTRACT**

**Contract Partners**:

Partner 1: Last and First Names: …………………………………

Residence / Location …………………………………

Contact Number: …………………………………

Function: …………………………………

Partner 2: Last and First Names: …………………………………

Residence / Location: …………………………………

Contact Number: …………………………………

Function: …………………………………

Both parties agree to undertake (respect) the following clauses:

Clause 1: Partner 1 is the initiator of the present contract

Clause 2: Both parties must respect the contract

Clause 3: Partner 1 agrees to buy ……………kilograms of rice paddy produced by Partner 2

Clause 4: Partner 1 will not provide any input to the production by Partner 2

Clause 5: Partner 1 is committed to training Partner 2 on the following topics: agricultural contracts, rice production techniques, farm management, and calculating the cost of rice production

Clause 6: Partner 2 commits to providing rice of the variety *IR841* to Partner 1

Clause 7: Partner 2 is committed to providing rice of ……… percent of impurities to Partner 1

Clause 8: Partner 2 agrees to sell paddy rice at …………..….FCFA/kilogram to Partner 1. Partner 1 agrees to buy rice paddy at …………..………….FCFA/kilogram

Clause 9: Partner 2 agrees to deliver the rice paddy in the month of ……….…………. in the year of ……………………..

Clause 10: Both partners commit to be faithful to their commitments.

Clause 11: The present contract will last …….......…..months from ...…/…../……

Clause 12: Delivery of the rice will be in the village group.

Clause 13: The packaging of rice paddy are lost or recoverable.

Clause 14: Paddy rice will be delivered in 100 kilogram bags for packaging of 80 kilogram.

Clause 15: the present contract is a contract: (fixed period/duration undetermined)

Clause 16: Payment for rice for Mr./Mrs. ……………… will……………… (*in kind* / in cash)

Clause 17: In case of conflict, the regulation will be in ………………………………… (friendly / court)

***Partner 1 Signature Partner 2 Signature***

***First and last name First and last name***

***Witnesses***

***First and last name First and last name***

***Made in …………………the …/……/201……..***

Object of Contract: Production of rice paddy by ………………………………… for the delivery to …………………………………

**CONTRACT**

**Contract Partners**:

Partner 1: Last and First Names: …………………………………

Residence / Location …………………………………

Contact Number: …………………………………

Function: …………………………………

Partner 2: Last and First Names: …………………………………

Residence / Location: …………………………………

Contact Number: …………………………………

Function: …………………………………

Both parties agree to undertake (respect) the following clauses:

Clause 1: Partner 1 is the initiator of the present contract

Clause 2: Both parties must respect the contract

Clause 3: Partner 1 agrees to buy ……………kilograms of rice paddy produced by Partner 2

Clause 4: Partner 1 is committed to providing seed (……..……….kilograms) and fertilizer (……..……….kilograms) for Partner 2

Clause 5: Partner 1 is committed to training Partner 2 on the following topics: agricultural contracts, rice production techniques, farm management, and calculating the cost of rice production

Clause 6: Partner 2 commits to providing rice of the variety *IR841* to Partner 1

Clause 7: Partner 2 is committed to providing rice of ……… percent of impurities to Partner 1

Clause 8: Partner 2 agrees to sell paddy rice at …………..….FCFA/kilogram to Partner 1. Partner 1 agrees to buy rice paddy at …………..………….FCFA/kilogram

Clause 9: Partner 2 agrees to deliver the rice paddy in the month of ……….…………. in the year of ……………………..

Clause 10: Both partners commit to be faithful to their commitments.

Clause 11: The present contract will last …….......…..months from ...…/…../……

Clause 12: Delivery of the rice will be in the village group.

Clause 13: The packaging of rice paddy are lost or recoverable.

Clause 14: Paddy rice will be delivered in 100 kilogram bags for packaging of 80 kilogram.

Clause 15: the present contract is a contract: (fixed period/duration undetermined)

Clause 16: Payment for rice for Mr./Mrs. ……………… will……………… (*in kind* / in cash)

Clause 17: In case of conflict, the regulation will be in ………………………………… (friendly / court)

***Partner 1 Signature Partner 2 Signature***

***First and last name First and last name***

***Witnesses***

***First and last name First and last name***

***Made in …………………the …/……/201……..***

## **Appendix B: Timeline and Robustness checks**

In this appendix we present a timeline of the experiment plus the full results from the various robustness checks referenced in the paper.

*Figure B.1: Timeline of experiment and rice growing season*

| Month/year | January 2016 | June  2016 | July  2016 | August  2016 | September 2016 | October 2016 | November 2016 | December 2016 | January 2017 |
| --- | --- | --- | --- | --- | --- | --- | --- | --- | --- |
| Activities of the RCT |  | Farmer group formation | Baseline data collection | Randomization, contracts signed with ESOP | Extension advice for T2 and T3,  Inputs for T3 | Extension advice for T2 and T3 | Mid-line data collection | Extension advice for T2 and T3 | End-line data collection |
| Rice growing season | Harvest |  |  |  | Sowing |  |  |  | Harvest |

Table B.1: Coefficient of variation in outcome variables by treatment

|  | Control (n=220) | Price [T1] (n=112) | Extension & price [T2] (n=255) | Input loans, extension, & price [T3] (n=268) |
| --- | --- | --- | --- | --- |
| Rice area (ha) | 0.464 | 0.418 | 0.444 | 0.415 |
| Yield (kg/ha) | 0.416 | 0.392 | 0.423 | 0.455 |
| Market participation (%) | 0.545 | 0.336 | 0.276 | 0.267 |
| Income per capita (US$) | 1.376 | 1.059 | 0.890 | 0.904 |
| *Note*: Table reports the coefficient of variation by treatment and by outcome variable. | | | | |

*Table B.2: Regression of attrition on treatment*

|  | (1) | (2) | (3) |
| --- | --- | --- | --- |
| Treatment (=1) | 0.033*** | 0.026 | 0.025 |
|  | (0.011) | (0.021) | (0.025) |
| Observations | 953 | 953 | 953 |
| R-squared | 0.015 | 0.076 | 0.089 |
| Arrondissement FE | No | Yes | Yes |
| Household covariates | No | No | Yes |

Note: Columns present OLS regressions of attrition on treatment. Column 1 contains only treatment as an independent variable, column 2 adds arrondissement fixed effects, and column 3 adds household covariates. Covariates include household size, age and gender of household head, number of years growing rice, and indicators for if the household head had at least primary education, if farming is the household’s main activity, if they have received extension training previously, and if they are a member of a farmer association. Standard errors clustered at the farmer-group-level are in parentheses (*** $p<0.01$, ** $p<0.05$, * $p<0.10$).

Table B.3: Treatment effects of farming contract on inputs [T-C]

|  | ln(seed)  (CFA/ha) | ln(fertilizer)  (kg/ha) | ln(pesticide)  (CFA/ha) | ln(herbicide)  (CFA/ha) | ln(labor)  (days/ha) |
| --- | --- | --- | --- | --- | --- |
|  | (1) | (2) | (3) | (4) | (5) |
| Treatment effect | 1.326*** | 1.630*** | 0.516* | 1.095* | 0.956*** |
|  | (0.249) | (0.461) | (0.282) | (0.626) | (0.228) |
| Mean dependent variable in control | 3.148 | 2.608 | 0.005 | 1.819 | 2.156 |
| Observations | 855 | 855 | 855 | 855 | 855 |
| R-squared | 0.450 | 0.270 | 0.280 | 0.264 | 0.453 |
| Arrondissement FE | Yes | Yes | Yes | Yes | Yes |
| Household covariates | Yes | Yes | Yes | Yes | Yes |

*Note*: Columns present ANCOVA regressions. For simplicity, coefficient estimates are only reported for the treatment effect. Covariates include household size, age and gender of household head, number of years growing rice, and indicators for if the household head had at least primary education, if farming is the household’s main activity, if they have received extension training previously, and if they are a member of a farmer association. Standard errors clustered at the farmer-group-level are in parentheses (***** $p<0.01$*, *** $p<0.05$*, ** $p<0.1$).

Table B.4: Treatment effects of each contract characteristic on inputs [T3-T2-T1-C]

|  | ln(seed)  (CFA/ha) | ln(fertilizer)  (kg/ha) | ln(pesticide)  (CFA/ha) | ln(herbicide)  (CFA/ha) | ln(labor)  (days/ha) |
| --- | --- | --- | --- | --- | --- |
|  | (1) | (2) | (3) | (4) | (5) |
| Treatment effect of T1 | 1.412*** | 1.587*** | 0.666 | 0.966 | 0.944*** |
|  | (0.356) | (0.584) | (0.428) | (0.741) | (0.273) |
| Treatment effect of T2 | 1.341*** | 1.556*** | 0.520* | 0.836 | 0.844*** |
|  | (0.245) | (0.472) | (0.276) | (0.677) | (0.234) |
| Treatment effect of T3 | 1.289*** | 1.728*** | 0.475 | 1.433** | 1.080*** |
|  | (0.301) | (0.528) | (0.341) | (0.720) | (0.260) |
| Mean dependent variable in control | 3.148 | 2.608 | 0.005 | 1.819 | 2.156 |
| Observations | 855 | 855 | 855 | 855 | 855 |
| R-squared | 0.450 | 0.270 | 0.282 | 0.271 | 0.456 |
| Arrondissement FE | Yes | Yes | Yes | Yes | Yes |
| Household covariates | Yes | Yes | Yes | Yes | Yes |

*Note*: Columns present ANCOVA regressions. For simplicity, coefficient estimates are only reported for the treatment effect. Covariates include household size, age and gender of household head, number of years growing rice, and indicators for if the household head had at least primary education, if farming is the household’s main activity, if they have received extension training previously, and if they are a member of a farmer association. Standard errors clustered at the farmer-group-level are in parentheses (***** $p<0.01$*, *** $p<0.05$*, ** $p<0.10$).

Table B.5: Lee bounds on the treatment effects of farming contract [T-C]

|  | OLS | Lee Bounds | |
| --- | --- | --- | --- |
|  |  | Lower | Upper |
|  | (1) | (2) | (3) |
| Rice area (ha) | 0.199*** | 0.120*** | 0.292*** |
|  | (0.055) | (0.033) | (0.032) |
| Yield (kg/ha) | 466.9*** | 319.9*** | 641.1*** |
|  | (98.08) | (64.36) | (63.43) |
| Market participation (%) | 32.95*** | 32.15*** | 38.60*** |
|  | (2.634) | (1.274) | (1.271) |
| Income per capita (US$) | 120.0* | 109.7*** | 259.6*** |
|  | (66.51) | (32.26) | (20.96) |
| *Note*: Each row reports results from OLS regressions and corresponding Lee bounds. Column (1) reproduces the OLS results from the tables presented in the paper. Columns (2) and (3) reports lower and upper Lee bounds for the corresponding regression. | | | |

Table B.6: Correction for multiple inference of the treatment effects of farming contract [T-C]

|  | OLS | OLS | ANCOVA | ANCOVA |
| --- | --- | --- | --- | --- |
|  | (1) | (2) | (3) | (4) |
| Panel A: rice area (ha) |  |  |  |  |
| Unadjusted *p*-value | 0.0003 | 0.0024 | 0.0005 | 0.0024 |
| Bonferroni adjusted *p*-value | 0.0013 |  |  |  |
| Holm adjusted *p*-value | 0.0007 |  |  |  |
| List et al. adjusted *p*-value | 0.0003 |  |  |  |
| Sharpened *q*-value | 0.0010 | 0.0030 | 0.0010 | 0.0030 |
| Panel B: yield (kg/ha) |  |  |  |  |
| Unadjusted *p*-value | 0.0003 | 0.0000 | 0.0000 | 0.0000 |
| Bonferroni adjusted *p*-value | 0.0013 |  |  |  |
| Holm adjusted *p*-value | 0.0013 |  |  |  |
| List et al. adjusted *p*-value | 0.0003 |  |  |  |
| Sharpened *q*-value | 0.0010 | 0.0010 | 0.0010 | 0.0010 |
| Panel C: market participation (%) |  |  |  |  |
| Unadjusted *p*-value | 0.0003 | 0.0000 | 0.0000 | 0.0000 |
| Bonferroni adjusted *p*-value | 0.0013 |  |  |  |
| Holm adjusted *p*-value | 0.0010 |  |  |  |
| List et al. adjusted *p*-value | 0.0003 |  |  |  |
| Sharpened *q*-value | 0.0010 | 0.0010 | 0.0010 | 0.0010 |
| Panel D: income per capita (US$) |  |  |  |  |
| Unadjusted *p*-value | 0.0003 | 0.0827 | 0.0196 | 0.0434 |
| Bonferroni adjusted *p*-value | 0.0013 |  |  |  |
| Holm adjusted *p*-value | 0.0003 |  |  |  |
| List et al. adjusted *p*-value | 0.0003 |  |  |  |
| Sharpened *q*-value | 0.0830 | 0.0830 | 0.0790 | 0.0830 |
| *Note*: Each cell contains *p*- or *q*-values for the multiple regressions presented in Table 5. We correct for testing four hypotheses (four outcome variables and one treatment). Bonferroni, Holm, and List et al. adjusted *p*-values are calculated using the Stata code from List et al. (2019). The sharpened *q*-values are calculated using the Stata code from Anderson (2008). Note that the code in List et al. (2019) only makes adjustments for OLS estimates of the treatment effect. Additionally, the calculations do not accommodate the presence of covariates or other controls. | | | | |

Table B.7: Randomization inference p-values on the treatment effects of farming contract [T-C]

|  | OLS | OLS | ANCOVA | ANCOVA |
| --- | --- | --- | --- | --- |
|  | (1) | (2) | (3) | (4) |
| Rice area (ha) | | | | |
| Unadjusted *p*-value | 0.0005 | 0.0023 | 0.0005 | 0.0024 |
| RI adjusted *p*-value | 0.0280 | 0.0510 | 0.0290 | 0.0510 |
| Panel B: yield (kg/ha) | | | | |
| Unadjusted *p*-value | 0.0000 | 0.0000 | 0.0000 | 0.0000 |
| RI adjusted *p*-value | 0.0020 | 0.0010 | 0.0020 | 0.0000 |
| Panel C: market participation (%) | | | | |
| Unadjusted *p*-value | 0.0000 | 0.0000 | 0.0000 | 0.0000 |
| RI adjusted *p*-value | 0.0000 | 0.0000 | 0.0000 | 0.0000 |
| Panel D: income per capita (US$) | | | | |
| Unadjusted *p*-value | 0.0196 | 0.0832 | 0.0196 | 0.0437 |
| RI adjusted *p*-value | 0.0850 | 0.1120 | 0.0850 | 0.0810 |

*Note*: The table reports *p*-values adjusted using the randomization inference procedure outlined in Heß (2017). The *p*-values are calculated in relation to the distribution of treatment effect under the sharp null of no treatment effect from 1,000 random draws. The Table also reports *p*-values corresponding to the regression results presented in Table 5.

Table B.8: Correction for multiple inference of the treatment effects of each contract characteristic [T3-T2-T1-C]

|  |  | OLS | OLS | ANCOVA | ANCOVA |
| --- | --- | --- | --- | --- | --- |
|  |  | (1) | (2) | (3) | (4) |
| Panel A: rice area (ha) | |  |  |  |  |
| Treatment effect of T1 | Unadjusted *p*-value | 0.0005 | 0.0012 | 0.0005 | 0.0013 |
|  | Bonferroni adjusted *p*-value | 0.0040 |  |  |  |
|  | Holm adjusted *p*-value | 0.0023 |  |  |  |
|  | List et al. adjusted *p*-value | 0.0003 |  |  |  |
|  | Sharpened *q*-value | 0.0010 | 0.0020 | 0.0010 | 0.0020 |
| Treatment effect of T2 | Unadjusted *p*-value | 0.0062 | 0.0249 | 0.0067 | 0.0264 |
|  | Bonferroni adjusted *p*-value | 0.0040 |  |  |  |
|  | Holm adjusted *p*-value | 0.0017 |  |  |  |
|  | List et al. adjusted *p*-value | 0.0003 |  |  |  |
|  | Sharpened *q*-value | 0.0070 | 0.0240 | 0.0070 | 0.0240 |
| Treatment effect of T3 | Unadjusted *p*-value | 0.0001 | 0.0003 | 0.0001 | 0.0003 |
|  | Bonferroni adjusted *p*-value | 0.0040 |  |  |  |
|  | Holm adjusted *p*-value | 0.0027 |  |  |  |
|  | List et al. adjusted *p*-value | 0.0003 |  |  |  |
|  | Sharpened *q*-value | 0.0010 | 0.0010 | 0.0010 | 0.0010 |
| Panel B: yield (kg/ha) | |  |  |  |  |
| Treatment effect of T1 | Unadjusted *p*-value | 0.0001 | 0.0006 | 0.0001 | 0.0008 |
|  | Bonferroni adjusted *p*-value | 0.0040 |  |  |  |
|  | Holm adjusted *p*-value | 0.0010 |  |  |  |
|  | List et al. adjusted *p*-value | 0.0003 |  |  |  |
|  | Sharpened *q*-value | 0.0010 | 0.0010 | 0.0010 | 0.0020 |
| Treatment effect of T2 | Unadjusted *p*-value | 0.0001 | 0.0001 | 0.0001 | 0.0001 |
|  | Bonferroni adjusted *p*-value | 0.0040 |  |  |  |
|  | Holm adjusted *p*-value | 0.0040 |  |  |  |
|  | List et al. adjusted *p*-value | 0.0003 |  |  |  |
|  | Sharpened *q*-value | 0.0010 | 0.0010 | 0.0010 | 0.0010 |
| Treatment effect of T3 | Unadjusted *p*-value | 0.0000 | 0.0000 | 0.0000 | 0.0000 |
|  | Bonferroni adjusted *p*-value | 0.0040 |  |  |  |
|  | Holm adjusted *p*-value | 0.0013 |  |  |  |
|  | List et al. adjusted *p*-value | 0.0003 |  |  |  |
|  | Sharpened *q*-value | 0.0010 | 0.0010 | 0.0010 | 0.0010 |
| Panel C: market participation (%) | |  |  |  |  |
| Treatment effect of T1 | Unadjusted *p*-value | 0.0000 | 0.0000 | 0.0000 | 0.0000 |
|  | Bonferroni adjusted *p*-value | 0.0040 |  |  |  |
|  | Holm adjusted *p*-value | 0.0037 |  |  |  |
|  | List et al. adjusted *p*-value | 0.0003 |  |  |  |
|  | Sharpened *q*-value | 0.0010 | 0.0010 | 0.0010 | 0.0010 |
| Treatment effect of T2 | Unadjusted *p*-value | 0.0000 | 0.0000 | 0.0000 | 0.0000 |
|  | Bonferroni adjusted *p*-value | 0.0040 |  |  |  |
|  | Holm adjusted *p*-value | 0.0020 |  |  |  |
|  | List et al. adjusted *p*-value | 0.0003 |  |  |  |
|  | Sharpened *q*-value | 0.0010 | 0.0010 | 0.0460 | 0.0010 |
| Treatment effect of T3 | Unadjusted *p*-value | 0.0000 | 0.0000 | 0.0000 | 0.0000 |
|  | Bonferroni adjusted *p*-value | 0.0040 |  |  |  |
|  | Holm adjusted *p*-value | 0.0033 |  |  |  |
|  | List et al. adjusted *p*-value | 0.0003 |  |  |  |
|  | Sharpened *q*-value | 0.0010 | 0.0010 | 0.0010 | 0.0010 |
| Panel D: income per capita (US$) | |  |  |  |  |
| Treatment effect of T1 | Unadjusted *p*-value | 0.0718 | 0.2154 | 0.0401 | 0.1555 |
|  | Bonferroni adjusted *p*-value | 0.1760 |  |  |  |
|  | Holm adjusted *p*-value | 0.0147 |  |  |  |
|  | List et al. adjusted *p*-value | 0.0147 |  |  |  |
|  | Sharpened *q*-value | 0.1530 | 0.2530 | 0.0880 | 0.2100 |
| Treatment effect of T2 | Unadjusted *p*-value | 0.2797 | 0.2302 | 0.1054 | 0.1489 |
|  | Bonferroni adjusted *p*-value | 0.0240 |  |  |  |
|  | Holm adjusted *p*-value | 0.0040 |  |  |  |
|  | List et al. adjusted *p*-value | 0.0037 |  |  |  |
|  | Sharpened *q*-value | 0.2710 | 0.2530 | 0.2840 | 0.2100 |
| Treatment effect of T3 | Unadjusted *p*-value | 0.0141 | 0.0168 | 0.0022 | 0.0064 |
|  | Bonferroni adjusted *p*-value | 0.0040 |  |  |  |
|  | Holm adjusted *p*-value | 0.0030 |  |  |  |
|  | List et al. adjusted *p*-value | 0.0003 |  |  |  |
|  | Sharpened *q*-value | 0.0570 | 0.0590 | 0.0280 | 0.0460 |
| *Note*: Each cell contains *p*- or *q*-values for the multiple regressions presented in Table 6. We correct for testing twelve hypotheses (four outcome variables and three treatments). Bonferroni, Holm, and List et al. adjusted *p*-values are calculated using the Stata code from List et al. (2019). The sharpened *q*-values are calcualted using the Stata code from Anderson (2008). Note that the code in List et al. (2019) only makes adjustments for OLS estimates of the treatment effect. Additionally, the calculations do not accommodate the presence of covariates or other controls. | | | | | |

Table B.9: Randomization inference p-values on the treatment effects of each contract characteristic [T3-T2-T1-C]

|  |  | | OLS | | OLS | | ANCOVA | | ANCOVA | |
| --- | --- | --- | --- | --- | --- | --- | --- | --- | --- | --- |
|  |  | | (1) | | (2) | | (3) | | (4) | |
| Panel A: rice area (ha) | |  | |  | |  | |  | |  |
| T1 | Unadjusted *p*-value | | 0.0005 | | 0.0012 | | 0.0005 | | 0.0013 | |
|  | RI adjusted *p*-value | | 0.1220 | | 0.2530 | | 0.1220 | | 0.2530 | |
| T2 | Unadjusted *p*-value | | 0.0062 | | 0.0249 | | 0.0067 | | 0.0264 | |
|  | RI adjusted *p*-value | | 0.0070 | | 0.0150 | | 0.0080 | | 0.0200 | |
| T3 | Unadjusted *p*-value | | 0.0001 | | 0.0003 | | 0.0001 | | 0.0003 | |
|  | RI adjusted *p*-value | | 0.0230 | | 0.0300 | | 0.0230 | | 0.0330 | |
| Panel B: yield (kg/ha) | |  | |  | |  | |  | |  |
| T1 | Unadjusted *p*-value | | 0.0001 | | 0.0006 | | 0.0001 | | 0.0008 | |
|  | RI adjusted *p*-value | | 0.0300 | | 0.0300 | | 0.0300 | | 0.0300 | |
| T2 | Unadjusted *p*-value | | 0.0001 | | 0.0001 | | 0.0001 | | 0.0001 | |
|  | RI adjusted *p*-value | | 0.0010 | | 0.0010 | | 0.0010 | | 0.0010 | |
| T3 | Unadjusted *p*-value | | 0.0000 | | 0.0000 | | 0.0000 | | 0.0000 | |
|  | RI adjusted *p*-value | | 0.0001 | | 0.0001 | | 0.0001 | | 0.0001 | |
| Panel C: market participation (%) | |  | |  | |  | |  | |  |
| T1 | Unadjusted *p*-value | | 0.0000 | | 0.0000 | | 0.0000 | | 0.0000 | |
|  | RI adjusted *p*-value | | 0.0030 | | 0.0030 | | 0.0030 | | 0.0300 | |
| T2 | Unadjusted *p*-value | | 0.0000 | | 0.0000 | | 0.0000 | | 0.0000 | |
|  | RI adjusted *p*-value | | 0.0004 | | 0.0004 | | 0.0004 | | 0.0004 | |
| T3 | Unadjusted *p*-value | | 0.0000 | | 0.0000 | | 0.0000 | | 0.0000 | |
|  | RI adjusted *p*-value | | 0.0000 | | 0.0000 | | 0.0000 | | 0.0000 | |
| Panel D: income per capita (US$) | |  | |  | |  | |  | |  |
| T1 | Unadjusted *p*-value | | 0.0718 | | 0.2154 | | 0.0401 | | 0.1555 | |
|  | RI adjusted *p*-value | | 0.5680 | | 0.2880 | | 0.1410 | | 0.1820 | |
| T2 | Unadjusted *p*-value | | 0.2797 | | 0.2302 | | 0.1054 | | 0.1489 | |
|  | RI adjusted *p*-value | | 0.5180 | | 0.3340 | | 0.2140 | | 0.2280 | |
| T3 | Unadjusted *p*-value | | 0.0141 | | 0.0168 | | 0.0022 | | 0.0064 | |
|  | RI adjusted *p*-value | | 0.0680 | | 0.0450 | | 0.0420 | | 0.0540 | |
| *Note*: The table reports *p*-values adjusted using the randomization inference procedure outlined in Heß (2017). The *p*-values are calculated in relation to the distribution of treatment effect under the sharp null of no treatment effect from 1,000 random draws. The Table also reports *p*-values corresponding to the regression results presented in Table 6. | | | | | | | | | |  |

Table B.10: Treatment effects of price guarantee [T1-C]

|  | OLS | OLS | ANCOVA | ANCOVA |
| --- | --- | --- | --- | --- |
|  | (1) | (2) | (3) | (4) |
| Panel A: rice area (ha) |  |  |  |  |
| Treatment effect | 0.139** | 0.041 | 0.140** | 0.040 |
|  | (0.064) | (0.093) | (0.066) | (0.094) |
| Mean dependent variable in control | 0.772 | | | |
| Observations | 333 | 333 | 333 | 333 |
| R-squared | 0.126 | 0.146 | 0.126 | 0.147 |
| Arrondissement FE | Yes | Yes | Yes | Yes |
| Household covariates | No | Yes | No | Yes |
| Panel B: yield (kg/ha) |  |  |  |  |
| Treatment effect | 603.9*** | 459.1*** | 601.5*** | 450.6*** |
|  | (144.7) | (160.7) | (143.8) | (158.1) |
| Mean dependent variable in control | 1,652 | | | |
| Observations | 333 | 333 | 333 | 333 |
| R-squared | 0.159 | 0.186 | 0.159 | 0.186 |
| Arrondissement FE | Yes | Yes | Yes | Yes |
| Household covariates | No | Yes | No | Yes |
| Panel C: market participation (%) |  |  |  |  |
| Treatment effect | 20.36*** | 20.25*** | 19.59** | 19.58*** |
|  | (2.407) | (2.773) | (2.400) | (2.706) |
| Mean dependent variable in control | 24.96 | | | |
| Observations | 333 | 333 | 333 | 333 |
| R-squared | 0.484 | 0.494 | 0.486 | 0.495 |
| Arrondissement FE | Yes | Yes | Yes | Yes |
| Household covariates | No | Yes | No | Yes |
| Panel D: income per capita (US$) |  |  |  |  |
| Treatment effect | -10.66 | -14.98 | 73.98* | 13.54 |
|  | (27.59) | (50.06) | (37.91) | (44.09) |
| Mean dependent variable in control | 60.70 | | | |
| Observations | 333 | 333 | 333 | 333 |
| R-squared | 0.140 | 0.337 | 0.260 | 0.387 |
| Arrondissement FE | Yes | Yes | Yes | Yes |
| Household covariates | No | Yes | No | Yes |

*Note*: For simplicity, coefficient estimates are only reported for the treatment effect. Covariates include household size, age and gender of household head, number of years growing rice, and indicators for if the household head had at least primary education, if farming is the household’s main activity, if they have received extension training previously, and if they are a member of a farmer association. Standard errors clustered at the farmer-group-level are in parentheses (***** $p<0.01$*, *** $p<0.05$*, ** $p<0.10$).

Table B.11: Treatment effects of extension training and price guarantee [T2-C]

|  | OLS | OLS | ANCOVA | ANCOVA |
| --- | --- | --- | --- | --- |
|  | (1) | (2) | (3) | (4) |
| Panel A: rice area (ha) |  |  |  |  |
| Treatment effect | 0.140*** | 0.118** | 0.139*** | 0.117** |
|  | (0.040) | (0.046) | (0.041) | (0.047) |
| Mean dependent variable in control | 0.772 | | | |
| Observations | 475 | 475 | 475 | 475 |
| R-squared | 0.058 | 0.068 | 0.058 | 0.068 |
| Arrondissement FE | Yes | Yes | Yes | Yes |
| Household covariates | No | Yes | No | Yes |
| Panel B: yield (kg/ha) |  |  |  |  |
| Treatment effect | 443.9*** | 484.94*** | 440.26*** | 481.17*** |
|  | (97.47) | (78.86) | (97.95) | (77.59) |
| Mean dependent variable in control | 1,652 | | | |
| Observations | 475 | 475 | 475 | 475 |
| R-squared | 0.116 | 0.133 | 0.117 | 0.134 |
| Arrondissement FE | Yes | Yes | Yes | Yes |
| Household covariates | No | Yes | No | Yes |
| Panel C: market participation (%) |  |  |  |  |
| Treatment effect | 32.32*** | 32.05*** | 32.12*** | 31.87*** |
|  | (2.395) | (2.365) | (2.168) | (2.281) |
| Mean dependent variable in control | 24.96 | | | |
| Observations | 475 | 475 | 475 | 475 |
| R-squared | 0.587 | 0.598 | 0.591 | 0.601 |
| Arrondissement FE | Yes | Yes | Yes | Yes |
| Household covariates | No | Yes | No | Yes |
| Panel D: income per capita (US$) |  |  |  |  |
| Treatment effect | 121.2 | 111.5 | 166.9 | 126.3 |
|  | (94.71) | (88.19) | (100.8) | (89.72) |
| Mean dependent variable in control | 60.70 | | | |
| Observations | 475 | 475 | 475 | 475 |
| R-squared | 0.082 | 0.281 | 0.142 | 0.289 |
| Arrondissement FE | Yes | Yes | Yes | Yes |
| Household covariates | No | Yes | No | Yes |

*Note*: For simplicity, coefficient estimates are only reported for the treatment effect. Covariates include household size, age and gender of household head, number of years growing rice, and indicators for if the household head had at least primary education, if farming is the household’s main activity, if they have received extension training previously, and if they are a member of a farmer association. Standard errors clustered at the farmer-group-level are in parentheses (***** $p<0.01$*, *** $p<0.05$*, ** $p<0.10$).

Table B.12: Treatment effects of input loans, extension training, and price guarantee [T3-C]

|  | OLS | OLS | ANCOVA | ANCOVA |
| --- | --- | --- | --- | --- |
|  | (1) | (2) | (3) | (4) |
| Panel A: rice area (ha) |  |  |  |  |
| Treatment effect | 0.310*** | 0.284*** | 0.312*** | 0.288*** |
|  | (0.085) | (0.088) | (0.086) | (0.088) |
| Mean dependent variable in control | 0.772 | | | |
| Observations | 487 | 487 | 487 | 487 |
| R-squared | 0.132 | 0.139 | 0.133 | 0.140 |
| Arrondissement FE | Yes | Yes | Yes | Yes |
| Household covariates | No | Yes | No | Yes |
| Panel B: yield (kg/ha) |  |  |  |  |
| Treatment effect | 509.1*** | 505.1*** | 506.3*** | 500.7*** |
|  | (143.8) | (169.7) | (141.6) | (166.2) |
| Mean dependent variable in control | 1,652 | | | |
| Observations | 487 | 487 | 487 | 487 |
| R-squared | 0.125 | 0.146 | 0.125 | 0.147 |
| Arrondissement FE | Yes | Yes | Yes | Yes |
| Household covariates | No | Yes | No | Yes |
| Panel C: market participation (%) |  |  |  |  |
| Treatment effect | 37.75*** | 38.96*** | 37.75*** | 38.96*** |
|  | (2.905) | (2.755) | (2.918) | (2.760) |
| Mean dependent variable in control | 24.96 | | | |
| Observations | 487 | 487 | 487 | 487 |
| R-squared | 0.661 | 0.672 | 0.661 | 0.672 |
| Arrondissement FE | Yes | Yes | Yes | Yes |
| Household covariates | No | Yes | No | Yes |
| Panel D: income per capita (US$) |  |  |  |  |
| Treatment effect | 151.7* | 141.4** | 174.5** | 151.2** |
|  | (77.17) | (65.65) | (75.232=) | (64.38) |
| Mean dependent variable in control | 60.70 | | | |
| Observations | 487 | 487 | 487 | 487 |
| R-squared | 0.099 | 0.313 | 0.154 | 0.320 |
| Arrondissement FE | Yes | Yes | Yes | Yes |
| Household covariates | No | Yes | No | Yes |

*Note*: For simplicity, coefficient estimates are only reported for the treatment effect. Covariates include household size, age and gender of household head, number of years growing rice, and indicators for if the household head had at least primary education, if farming is the household’s main activity, if they have received extension training previously, and if they are a member of a farmer association. Standard errors clustered at the farmer-group-level are in parentheses (***** $p<0.01$*, *** $p<0.05$*, ** $p<0.10$).

Table B.13: Treatment effects of extension training [T2-T1]

|  | OLS | OLS | ANCOVA | ANCOVA |
| --- | --- | --- | --- | --- |
|  | (1) | (2) | (3) | (4) |
| Panel A: rice area (ha) |  |  |  |  |
| Treatment effect | -0.114** | -0.105* | -0.115** | -0.107* |
|  | (0.055) | (0.058) | (0.056) | (0.059) |
| Mean dependent variable in control | 1.006 | | | |
| Observations | 368 | 368 | 368 | 368 |
| R-squared | 0.036 | 0.050 | 0.038 | 0.051 |
| Arrondissement FE | Yes | Yes | Yes | Yes |
| Household Covariates | No | Yes | No | Yes |
| Panel B: yield (kg/ha) |  |  |  |  |
| Treatment effect | -183.2* | -144.3 | -167.9 | -131.6 |
|  | (103.0) | (115.4) | (103.4) | (114.1) |
| Mean dependent variable in control | 2,154 | | | |
| Observations | 368 | 368 | 368 | 368 |
| R-squared | 0.043 | 0.049 | 0.047 | 0.052 |
| Arrondissement FE | Yes | Yes | Yes | Yes |
| Household Covariates | No | Yes | No | Yes |
| Panel C: market participation (%) |  |  |  |  |
| Treatment effect | 10.81*** | 8.913*** | 10.74*** | 8.730*** |
|  | (2.238) | (2.025) | (2.212) | (2.026) |
| Mean dependent variable in control | 51.21 | | | |
| Observations | 368 | 368 | 368 | 368 |
| R-squared | 0.083 | 0.122 | 0.083 | 0.123 |
| Arrondissement FE | Yes | Yes | Yes | Yes |
| Household Covariates | No | Yes | No | Yes |
| Panel D: income per capita (USD$) |  |  |  |  |
| Treatment effect | -37.29 | 17.87 | -23.86 | 15.52 |
|  | (44.06) | (42.31) | (40.70) | (40.39) |
| Mean dependent variable in control | 385.1 | | | |
| Observations | 368 | 368 | 368 | 368 |
| R-squared | 0.162 | 0.324 | 0.273 | 0.381 |
| Arrondissement FE | Yes | Yes | Yes | Yes |
| Household Covariates | No | Yes | No | Yes |

*Note*: For simplicity, coefficient estimates are only reported for the treatment effect. Covariates include household size, age and gender of household head, number of years growing rice, and indicators for if the household head had at least primary education, if farming is the household’s main activity, if they have received extension training previously, and if they are a member of a farmer association. Standard errors clustered at the farmer-group-level are in parentheses (***** $p<0.01$*, *** $p<0.05$*, ** $p<0.10$).

Table B.14: Treatment effects of input loans [T3-T2]

|  | OLS | OLS | ANCOVA | ANCOVA |
| --- | --- | --- | --- | --- |
|  | (1) | (2) | (3) | (4) |
| Panel A: rice area (ha) |  |  |  |  |
| Treatment effect | 0.145*** | 0.144*** | 0.146*** | 0.147*** |
|  | (0.039) | (0.040) | (0.038) | (0.039) |
| Mean dependent variable in control | 0.900 | | | |
| Observations | 522 | 522 | 522 | 522 |
| R-squared | 0.047 | 0.054 | 0.048 | 0.055 |
| Arrondissement FE | Yes | Yes | Yes | Yes |
| Household Covariates | No | Yes | No | Yes |
| Panel B: yield (kg/ha) |  |  |  |  |
| Treatment effect | 83.16 | 77.48 | 79.77 | 71.96 |
|  | (84.25) | (83.39) | (81.90) | (81.08) |
| Mean dependent variable in control | 2,074 | | | |
| Observations | 522 | 522 | 522 | 522 |
| R-squared | 0.032 | 0.051 | 0.037 | 0.057 |
| Arrondissement FE | Yes | Yes | Yes | Yes |
| Household Covariates | No | Yes | No | Yes |
| Panel C: market participation (%) |  |  |  |  |
| Treatment effect | 7.828*** | 8.923*** | 7.858*** | 8.941*** |
|  | (1.581) | (1.726) | (1.589) | (1.729) |
| Mean dependent variable in control | 58.06 | | | |
| Observations | 522 | 522 | 522 | 522 |
| R-squared | 0.100 | 0.132 | 0.103 | 0.135 |
| Arrondissement FE | Yes | Yes | Yes | Yes |
| Household Covariates | No | Yes | No | Yes |
| Panel D: income per capita (USD$) |  |  |  |  |
| Treatment effect | 93.57* | 78.50** | 91.52* | 80.55** |
|  | (48.00) | (38.05) | (46.82) | (38.56) |
| Mean dependent variable in control | 370.0 | | | |
| Observations | 522 | 522 | 522 | 522 |
| R-squared | 0.068 | 0.270 | 0.111 | 0.279 |
| Arrondissement FE | Yes | Yes | Yes | Yes |
| Household Covariates | No | Yes | No | Yes |

*Note*: For simplicity, coefficient estimates are only reported for the treatment effect. Covariates include household size, age and gender of household head, number of years growing rice, and indicators for if the household head had at least primary education, if farming is the household’s main activity, if they have received extension training previously, and if they are a member of a farmer association. Standard errors clustered at the farmer-group-level are in parentheses (***** $p<0.01$*, *** $p<0.05$*, ** $p<0.10$).

Table B.15: Treatment effects of input loans and extension training [T3-T1]

|  | OLS | OLS | ANCOVA | ANCOVA |
| --- | --- | --- | --- | --- |
|  | (1) | (2) | (3) | (4) |
| Panel A: rice area (ha) |  |  |  |  |
| Treatment effect | 0.039 | 0.030 | 0.040 | 0.031 |
|  | (0.050) | (0.048) | (0.051) | (0.050) |
| Mean dependent variable in control | 1.006 | | | |
| Observations | 380 | 380 | 380 | 380 |
| R-squared | 0.025 | 0.036 | 0.025 | 0.036 |
| Arrondissement FE | Yes | Yes | Yes | Yes |
| Household Covariates | No | Yes | No | Yes |
| Panel B: yield (kg/ha) |  |  |  |  |
| Treatment effect | 84.38 | 73.01 | 79.76 | 68.49 |
|  | (103.9) | (113.4) | (103.4) | (112.7) |
| Mean dependent variable in control | 2,154 | | | |
| Observations | 380 | 380 | 380 | 380 |
| R-squared | 0.037 | 0.061 | 0.039 | 0.062 |
| Arrondissement FE | Yes | Yes | Yes | Yes |
| Household Covariates | No | Yes | No | Yes |
| Panel C: market participation (%) |  |  |  |  |
| Treatment effect | 17.06*** | 16.91*** | 16.86*** | 16.61*** |
|  | (1.964) | (1.981) | (1.888) | (1.883) |
| Mean dependent variable in control | 51.21 | | | |
| Observations | 380 | 380 | 380 | 380 |
| R-squared | 0.197 | 0.223 | 0.212 | 0.237 |
| Arrondissement FE | Yes | Yes | Yes | Yes |
| Household Covariates | No | Yes | No | Yes |
| Panel D: income per capita (USD$) |  |  |  |  |
| Treatment effect | 41.53 | 93.53** | 91.62* | 121.1*** |
|  | (50.92) | (40.91) | (48.71) | (43.38) |
| Mean dependent variable in control | 385.1 | | | |
| Observations | 380 | 380 | 380 | 380 |
| R-squared | 0.094 | 0.318 | 0.211 | 0.378 |
| Arrondissement FE | Yes | Yes | Yes | Yes |
| Household Covariates | No | Yes | No | Yes |

*Note*: For simplicity, coefficient estimates are only reported for the treatment effect. Covariates include household size, age and gender of household head, number of years growing rice, and indicators for if the household head had at least primary education, if farming is the household’s main activity, if they have received extension training previously, and if they are a member of a farmer association. Standard errors clustered at the farmer-group-level are in parentheses (***** $p<0.01$*, *** $p<0.05$*, ** $p<0.10$).

Table B.16: Treatment effects of farming contract on income [T-C]

|  | OLS | OLS | ANCOVA | ANCOVA |
| --- | --- | --- | --- | --- |
|  | (1) | (2) | (3) | (4) |
| Panel A: ln(rice income) |  |  |  |  |
| Treatment effect | 1.443*** | 1.435*** | 1.459*** | 1.443*** |
|  | (0.523) | (0.537) | (0.524) | (0.545) |
| Mean dependent variable in control | 10.24 | | | |
| Observations | 855 | 855 | 855 | 855 |
| R-squared | 0.101 | 0.118 | 0.101 | 0.118 |
| Arrondissement FE | Yes | Yes | Yes | Yes |
| Household Covariates | No | Yes | No | Yes |
| Panel B: ln(other farm income) |  |  |  |  |
| Treatment effect | -0.199 | -0.079 | -0.135 | -0.030 |
|  | (0.631) | (0.613) | (0.617) | (0.603) |
| Mean dependent variable in control | 12.74 | | | |
| Observations | 855 | 855 | 855 | 855 |
| R-squared | 0.105 | 0.122 | 0.121 | 0.134 |
| Arrondissement FE | Yes | Yes | Yes | Yes |
| Household Covariates | No | Yes | No | Yes |
| Panel C: ln(non-farm income) |  |  |  |  |
| Treatment effect | 1.156 | 1.441 | 0.625 | 0.910 |
|  | (1.235) | (1.289) | (1.217) | (1.274) |
| Mean dependent variable in control | 4.658 | | | |
| Observations | 855 | 855 | 855 | 855 |
| R-squared | 0.260 | 0.269 | 0.286 | 0.292 |
| Arrondissement FE | Yes | Yes | Yes | Yes |
| Household Covariates | No | Yes | No | Yes |

*Note*: For simplicity, coefficient estimates are only reported for the treatment effect. Covariates include household size, age and gender of household head, number of years growing rice, and indicators for if the household head had at least primary education, if farming is the household’s main activity, if they have received extension training previously, and if they are a member of a farmer association. Standard errors clustered at the farmer-group-level are in parentheses (***** $p<0.01$*, *** $p<0.05$*, ** $p<0.10$).

Table B.17: Treatment effects of each contract characteristic on income [T3-T2-T1-C]

|  | OLS | OLS | ANCOVA | ANCOVA |
| --- | --- | --- | --- | --- |
|  | (1) | (2) | (3) | (4) |
| Panel A: ln(rice income) |  |  |  |  |
| Treatment effect of T1 | 1.464** | 1.382** | 1.490** | 1.393** |
|  | (0.582) | (0.604) | (0.579) | (0.611) |
| Treatment effect of T2 | 1.401** | 1.453*** | 1.416*** | 1.460*** |
|  | (0.537) | (0.540) | (0.539) | (0.546) |
| Treatment effect of T3 | 1.491*** | 1.424** | 1.510*** | 1.432** |
|  | (0.539) | (0.562) | (0.542) | (0.571) |
| Mean dependent variable in control | 10.24 | | | |
| Observations | 855 | 855 | 855 | 855 |
| R-squared | 0.101 | 0.118 | 0.101 | 0.118 |
| Arrondissement FE | Yes | Yes | Yes | Yes |
| Household covariates | No | Yes | No | Yes |
| Panel B: ln(other farm income) |  |  |  |  |
| Treatment effect of T1 | -0.299 | -0.126 | -0.258 | -0.098 |
|  | (0.624) | (0.633) | (0.610) | (0.622) |
| Treatment effect of T2 | -0.060 | 0.031 | -0.007 | 0.067 |
|  | (0.635) | (0.604) | (0.633) | (0.606) |
| Treatment effect of T3 | -0.348 | -0.213 | -0.262 | -0.143 |
|  | (0.670) | (0.659) | (0.640) | (0.634) |
| Mean dependent variable in control | 12.74 | | | |
| Observations | 855 | 855 | 855 | 855 |
| R-squared | 0.107 | 0.123 | 0.122 | 0.135 |
| Arrondissement FE | Yes | Yes | Yes | Yes |
| Household covariates | No | Yes | No | Yes |
| Panel C: ln(non-farm income) |  |  |  |  |
| Treatment effect of T1 | 2.114 | 2.658* | 1.604 | 2.126 |
|  | (1.466) | (1.533) | (1.425) | (1.494) |
| Treatment effect of T2 | 0.703 | 0.908 | 0.191 | 0.426 |
|  | (1.273) | (1.308) | (1.237) | (1.285) |
| Treatment effect of T3 | 1.402 | 1.845 | 0.840 | 1.266 |
|  | (1.449) | (1.478) | (1.398) | (1.440) |
| Mean dependent variable in control | 4.658 | | | |
| Observations | 855 | 855 | 855 | 855 |
| R-squared | 0.264 | 0.275 | 0.289 | 0.297 |
| Arrondissement FE | Yes | Yes | Yes | Yes |
| Household covariates | No | Yes | No | Yes |

*Note*: For simplicity, coefficient estimates are only reported for the treatment effect. Covariates include household size, age and gender of household head, number of years growing rice, and indicators for if the household head had at least primary education, if farming is the household’s main activity, if they have received extension training previously, and if they are a member of a farmer association. Standard errors clustered at the farmer-group-level are in parentheses (***** $p<0.01$*, *** $p<0.05$*, ** $p<0.10$)

Table B.18: Treatment effects of farming contract on food security [T-C]

|  | OLS | OLS | ANCOVA | ANCOVA |
| --- | --- | --- | --- | --- |
|  | (1) | (2) | (3) | (4) |
| Panel A: Household Food Insecurity Access Scale | | | | |
| Treatment effect | -1.104 | -0.845 | -2.088** | -1.782* |
|  | (1.218) | (1.263) | (0.987) | (1.027) |
| Mean dependent variable in control | 6.923 | | | |
| Observations | 855 | 855 | 855 | 855 |
| R-squared | 0.202 | 0.239 | 0.310 | 0.334 |
| Arrondissement FE | Yes | Yes | Yes | Yes |
| Household covariates | No | Yes | No | Yes |
| Panel B: Food Consumption Score |  |  |  |  |
| Treatment effect | 10.22*** | 8.474*** | 9.877*** | 8.240*** |
|  | (2.537) | (2.376) | (2.421) | (2.310) |
| Mean dependent variable in control | 56.95 | | | |
| Observations | 855 | 855 | 855 | 855 |
| R-squared | 0.136 | 0.162 | 0.141 | 0.165 |
| Arrondissement FE | Yes | Yes | Yes | Yes |
| Household covariates | No | Yes | No | Yes |

*Note*: For simplicity, coefficient estimates are only reported for the treatment effect. Covariates include household size, age and gender of household head, number of years growing rice, and indicators for if the household head had at least primary education, if farming is the household’s main activity, if they have received extension training previously, and if they are a member of a farmer association. Standard errors clustered at the farmer-group-level are in parentheses (***** $p<0.01$*, *** $p<0.05$*, ** $p<0.10$).

Table B.19: Treatment effects of each contract characteristic on food security [T3-T2-T1-C]

|  | OLS | OLS | ANCOVA | ANCOVA |
| --- | --- | --- | --- | --- |
|  | (1) | (2) | (3) | (4) |
| Panel A: Household Food Insecurity Access Scale | | | | |
| Treatment effect of T1 | -1.080 | -0.788 | -2.064 | -1.767 |
|  | (1.876) | (1.887) | (1.804) | (1.828) |
| Treatment effect of T2 | -0.900 | -0.693 | -2.068** | -1.779* |
|  | (1.294) | (1.296) | (1.004) | (1.021) |
| Treatment effect of T3 | -1.385 | -1.059 | -2.122* | -1.790 |
|  | (1.429) | (1.504) | (1.196) | (1.260) |
| Mean dependent variable in control | 6.922 | | | |
| Observations | 855 | 855 | 855 | 855 |
| R-squared | 0.203 | 0.240 | 0.310 | 0.334 |
| Arrondissement FE | Yes | Yes | Yes | Yes |
| Household covariates | No | Yes | No | Yes |
| Panel B: Food Consumption Score |  |  |  |  |
| Treatment effect of T1 | 6.738* | 5.197 | 6.677* | 5.137 |
|  | (4.002) | (3.930) | (3.906) | (3.876) |
| Treatment effect of T2 | 10.39*** | 8.289*** | 9.981*** | 8.023*** |
|  | (2.777) | (2.600) | (2.719) | (2.575) |
| Treatment effect of T3 | 11.28*** | 9.511*** | 11.00*** | 9.313*** |
|  | (3.541) | (3.271) | (3.429) | (3.207) |
| Mean dependent variable in control | 56.95 | | | |
| Observations | 855 | 855 | 855 | 855 |
| R-squared | 0.142 | 0.167 | 0.146 | 0.169 |
| Arrondissement FE | Yes | Yes | Yes | Yes |
| Household covariates | No | Yes | No | Yes |

*Note*: For simplicity, coefficient estimates are only reported for the treatment effect. Covariates include household size, age and gender of household head, number of years growing rice, and indicators for if the household head had at least primary education, if farming is the household’s main activity, if they have received extension training previously, and if they are a member of a farmer association. Standard errors clustered at the farmer-group-level are in parentheses (***** $p<0.01$*, *** $p<0.05$*, ** $p<0.10$).

Table B.20: Treatment effects of farming contract on rice profits [T-C]

|  | OLS | OLS | ANCOVA | ANCOVA |
| --- | --- | --- | --- | --- |
|  | (1) | (2) | (3) | (4) |
| Panel A: ln(profit) (CFA/ha) – low wages |  |  |  |  |
| Treatment effect | 0.423 | 0.411 | 0.387 | 0.242 |
|  | (1.712) | (1.761) | (1.792) | (1.850) |
| Mean dependent variable in control | 7.813 | | | |
| Observations | 855 | 855 | 855 | 855 |
| R-squared | 0.078 | 0.113 | 0.079 | 0.113 |
| Arrondissement FE | Yes | Yes | Yes | Yes |
| Household Covariates | No | Yes | No | Yes |
| Panel B: ln(profit) (CFA/ha) – high wages |  |  |  |  |
| Treatment effect | -1.867 | -1.993 | -1.380 | -1.749 |
|  | (2.137) | (2.215) | (2.199) | (2.313) |
| Mean dependent variable in control | 7.087 | | | |
| Observations | 855 | 855 | 855 | 855 |
| R-squared | 0.128 | 0.162 | 0.131 | 0.163 |
| Arrondissement FE | Yes | Yes | Yes | Yes |
| Household Covariates | No | Yes | No | Yes |
| Panel C: ln(profit) (CFA/ha) – self-reported wages | | | | |
| Treatment effect | 2.012** | 1.880* | 1.917* | 1.769* |
|  | (0.938) | (0.975) | (0.984) | (1.033) |
| Mean dependent variable in control | 9.245 | | | |
| Observations | 855 | 855 | 855 | 855 |
| R-squared | 0.078 | 0.097 | 0.078 | 0.097 |
| Arrondissement FE | Yes | Yes | Yes | Yes |
| Household Covariates | No | Yes | No | Yes |

*Note*: For simplicity, coefficient estimates are only reported for the treatment effect. Covariates include household size, age and gender of household head, number of years growing rice, and indicators for if the household head had at least primary education, if farming is the household’s main activity, if they have received extension training previously, and if they are a member of a farmer association. Standard errors clustered at the farmer-group-level are in parentheses (***** $p<0.01$*, *** $p<0.05$*, ** $p<0.10$).

Table B.21: Treatment effects of each contract characteristic on rice profits [T3-T2-T1-C]

|  | OLS | OLS | ANCOVA | ANCOVA |
| --- | --- | --- | --- | --- |
|  | (1) | (2) | (3) | (4) |
| Panel A: ln(profit) (CFA/ha) – low wages |  |  |  |  |
| Treatment effect of T1 | 1.166 | 0.884 | 1.149 | 0.691 |
|  | (2.063) | (2.157) | (2.133) | (2.246) |
| Treatment effect of T2 | 0.462 | 0.654 | 0.448 | 0.499 |
|  | (1.789) | (1.780) | (1.855) | (1.857) |
| Treatment effect of T3 | 0.090 | -0.023 | 0.077 | -0.159 |
|  | (1.818) | (1.868) | (1.899) | (1.957) |
| Mean dependent variable in control | 7.813 | | | |
| Observations | 855 | 855 | 855 | 855 |
| R-squared | 0.080 | 0.114 | 0.080 | 0.115 |
| Arrondissement FE | Yes | Yes | Yes | Yes |
| Household covariates | No | Yes | No | Yes |
| Panel B: ln(profit) (CFA/ha) – high wages |  |  |  |  |
| Treatment effect of T1 | -0.117 | -0.426 | 0.510 | -0.070 |
|  | (2.635) | (2.745) | (2.685) | (2.842) |
| Treatment effect of T2 | -1.683 | -1.552 | -1.104 | -1.233 |
|  | (2.223) | (2.231) | (2.267) | (2.320) |
| Treatment effect of T3 | -2.773 | -2.951 | -2.291 | -2.681 |
|  | (2.298) | (2.365) | (2.345) | (2.449) |
| Mean dependent variable in control | 7.087 | | | |
| Observations | 855 | 855 | 855 | 855 |
| R-squared | 0.134 | 0.168 | 0.138 | 0.169 |
| Arrondissement FE | Yes | Yes | Yes | Yes |
| Household covariates | No | Yes | No | Yes |
| Panel C: ln(profit) (CFA/ha) – self-reported wages | | | | |
| Treatment effect of T1 | 1.920** | 1.783** | 1.843** | 1.577* |
|  | (0.817) | (0.833) | (0.821) | (0.842) |
| Treatment effect of T2 | 2.009** | 2.056*** | 1.988** | 1.981*** |
|  | (0.766) | (0.754) | (0.760) | (0.744) |
| Treatment effect of T3 | 1.977** | 1.859** | 1.788** | 1.616** |
|  | (0.767) | (0.780) | (0.760) | (0.775) |
| Mean dependent variable in control | 9.245 | | | |
| Observations | 855 | 855 | 773 | 773 |
| R-squared | 0.097 | 0.113 | 0.096 | 0.112 |
| Arrondissement FE | Yes | Yes | Yes | Yes |
| Household covariates | No | Yes | No | Yes |

*Note*: For simplicity, coefficient estimates are only reported for the treatment effect. Covariates include household size, age and gender of household head, number of years growing rice, and indicators for if the household head had at least primary education, if farming is the household’s main activity, if they have received extension training previously, and if they are a member of a farmer association. Standard errors clustered at the farmer-group-level are in parentheses (***** $p<0.01$*, *** $p<0.05$*, ** $p<0.10$).
